# Exposure to socioeconomic adversity in early life and risk of depression at 18 years: The mediating role of locus of control

**DOI:** 10.1016/j.jad.2015.05.030

**Published:** 2015-09-01

**Authors:** Iryna Culpin, Lexine Stapinski, Ömür Budanur Miles, Ricardo Araya, Carol Joinson

**Affiliations:** a School of Social and Community Medicine, University of Bristol, Bristol, UK; b Centre of Research Excellence in Mental Health and Substance Use, University of New South Wales, Sydney, Australia; c Child and Adolescent Mental Health Service, St. David's Hospital Cardiff, Cwm Taf Health Board, Cardiff, Wales, UK; d London School of Hygiene & Tropical Medicine, Faculty of Epidemiology and Public Health, London, UK

**Keywords:** Avon Longitudinal Study of Parents and Children (ALSPAC), Early childhood, Socioeconomic adversity, Depression, Locus of control

## Abstract

**Background:**

Previous studies have linked exposure to early socioeconomic adversity to depression, but the mechanisms of this association are not well understood. Locus of control (LoC), an individual's control-related beliefs, has been implicated as a possible mechanism, however, longitudinal evidence to support this is lacking.

**Methods:**

The study sample comprised 8803 participants from a UK cohort, the Avon Longitudinal Study of Parents and Children (ALSPAC). Indicators of early socioeconomic adversity were collected from the antenatal period to 5 years and modelled as a latent factor. Depression was assessed using the Clinical Interview Schedule-Revised (CIS-R) at 18 years. LoC was assessed with the Nowicki–Strickland Internal–External (CNSIE) scale at 16 years.

**Results:**

Using structural equation modelling, we found that 34% of the total estimated association between early socioeconomic adversity and depression at 18 years was explained by external LoC at 16 years. There was weak evidence of a direct pathway from early socioeconomic adversity to depression after accounting for the indirect effect via external locus of control. Socioeconomic adversity was associated with more external LoC, which, in turn, was associated with depression.

**Limitations:**

Attrition may have led to an underestimation of the direct and indirect effect sizes in the complete case analysis.

**Conclusions:**

Results suggest that external LoC in adolescence is one of the factors mediating the link between early adversity and depression at 18 years. Cognitive interventions that seek to modify maladaptive control beliefs in adolescence may be effective in reducing risk of depression following early life adversity.

## Highlights

• We examine locus of control as a pathway between social adversity and depression.
• External locus of control mediates the link between social adversity and depression.
• Social adversity is linked to more external locus of control and depression.
• More external locus of control is associated with higher risk of depression.
• Depression prevention programs should address cognitive beliefs about control.

## 1 Introduction

An increasing body of research supports the association between early socioeconomic adversity and risk for depression in adolescence and young adulthood (Chapman et al., 2004; Kessler et al., 2010; Patten et al., 2014). In particular, socioeconomic disadvantage, poverty, inadequate housing, and residential instability during early childhood have been linked to later depression (Gilman et al., 2003, 2002). However, little is known about the psychological mechanisms underlying this association (Grant et al., 2006; Grant et al., 2003). Increased knowledge of factors explaining the link between aspects of early socioeconomic adversity and increased risk of depression could provide insights into potentially modifiable targets for intervention.

Depression is a complex disorder and a number of risk factors and causal mechanisms (e.g., psychosocial, neurocognitive, and gene–environment interplay) are likely to be involved (Maughan et al., 2013). Early socioeconomic adversity could exert a direct effect on depression via biological systems, such as the hypothalamic pituitary axis (HPA), and these effects could be independent of exposure to adulthood adversity (Stansfeld et al., 2011). Alternatively, early socioeconomic disadvantage may set off a chain of proximal psychosocial events and individual characteristics that increase the risk for depression, such as adverse family processes (e.g., negative parenting; Conger et al., 2002), environmental stressors (e.g., inadequate schooling; Barrera et al., 2002), and maladaptive coping styles and cognitive attributions (Paschall and Hubbard, 1998). Specifically, exposure to early socioeconomic adversity may lead to a pattern of psychological vulnerability characterised by maladaptive perceptions of the self and life events that increase risk for depression (Hammen, 2005). One aspect of psychological vulnerability that could be influential in the link between childhood adversity and depression is adolescents' internal versus external control-related beliefs also known as locus of control (LoC; Rotter, 1966; Strickland, 1989).

It has been argued that an individual's beliefs related to their perceived sense of control over their environment relate to their psychological well-being and mental health outcomes (Chorpita and Barlow, 1998; Shapiro et al., 1993). Individuals are thought to differ in the extent to which they perceive themselves as being able to control life events through their efforts and actions (internal LoC), or that life events are controlled by external forces such as luck, chance and powerful others (external LoC; Rotter, 1966). Although antecedents of LoC in adolescence are not fully understood, it has been suggested that early experiences of adverse and uncontrollable events, including persistent exposure to socioeconomic disadvantage, may foster external LoC orientation characterised by diminished sense of perceived control over one's life and environment (Bryant and Trockel, 1976; Chorpita, 2001; Gilman et al., 2003). Children and adolescents who develop external LoC and experience uncertainty about the extent of control they have over life events have also been hypothesised to be at increased risk of developing depression (Chorpita, 2001; Ostrander and Herman, 2006).

Socioeconomic differences in the sense of personal control have been examined in early cross-sectional studies (Lachman and Weaver, 1998), indicating that those in more disadvantaged groups (characterised by lower income and less education) have lower sense of control and are more likely to believe in the role of external forces and powerful others (Bosma et al., 1999; Lachman and Weaver, 1998). However, longitudinal evidence linking early socioeconomic adversity and adolescent LoC orientation is lacking. Similarly, numerous cross-sectional but few longitudinal studies have examined the link between LoC orientation and depression. Consistently, an external LoC has been found to be associated with depression in childhood (Cole et al., 2001), adolescence (Donnelly, 1999; Muris et al., 2004) and adulthood (Benassi et al., 1988). However, prospective longitudinal studies examining the association between external LoC and depression are limited (Harrow et al., 2009; Frenkel et al., 1995) and further investigations are warranted.

Studies that examine LoC as a possible pathway in the early adversity–depression association are also scarce and not without limitations. The majority of studies are cross-sectional and rely on retrospective assessment of childhood adversity and LoC, thus precluding inferences about the temporal relationship among experiences of adversity, LoC orientation and depression (Deardorff et al., 2003; Kim et al., 1997; Sandler et al., 2000). Furthermore, these studies focus on examining the possible mediating role of LoC in specific samples of children such as those from divorced, bereaved or severely disadvantaged families (Deardorff et al., 2003; Haine et al., 2003; Kim et al., 1997; Sandler et al., 2000). Other limitations include overreliance on measures based on a single reporter (e.g., adolescent self-reports; Kim et al., 1997), composite measures of stress (e.g., total number of various negative life events; Kim et al., 1997), and lack of diagnostic measures of depression (Deardorff et al., 2003). Thus, there is need for prospective studies to examine possible mediating role of LoC in the association between exposure to various aspects of early socioeconomic adversity and depression in young adulthood.

Family adverse experiences are multifaceted and dynamic. Thus, it is important to control for possible confounders whilst examining the pathways among early socioeconomic adversity, LoC and depression in young adulthood. For instance, indices of socioeconomic disadvantage, such as poverty, often co-occur with parental depression and negative parental cognitions (Edwards et al., 2003; Dong et al., 2004), and these events are associated with both development of external LoC orientation and depression in young adulthood. Epidemiological evidence has long established a strong link between socioeconomic disadvantage in childhood and increased risk of a psychiatric disorder, including depression (Solantaus et al., 2004). Paternal depression, like maternal depression, may compromise parenting behaviours and have an adverse impact on the way parents interact with their children (Lyons-Ruth et al., 2002; Paulson et al., 2006) who are more likely to develop external LoC. For instance, aspects of parental cognition, especially maternal warmth and acceptance, have been linked to internal LoC orientation in children and are considered to be antecedents of LoC (Carton and Nowicki, 1996; Muris et al., 2004).

The current study, using data from the Avon Longitudinal Study of Parents and Children (ALSPAC), examines whether LoC mediates the association between early socioeconomic adversity and later depression. It has been previously demonstrated in this cohort that aspects of childhood adversity such as victimisation (e.g., bullying) and harsh parenting are associated with more external LoC orientation (Fisher et al., 2013), and other studies have also reported an association between exposure to socioeconomic adversity and increased risk of depression (Joinson et al., unpublished results). We hypothesised that exposure to socioeconomic adversity from birth to 5 years will be associated with more external LoC orientation at 16 years and that this would constitute an indirect pathway between early adversity and increased risk of depression at 18 years. We used structural equation modelling (SEM) to test the hypothesised model using a latent factor to encapsulate exposure to early socioeconomic adversity during the first 5 years of life, and by adjusting the model for a range of child and parental confounders.

## 2 Method

### 2.1 Participants

The sample is comprised of participants from the Avon Longitudinal Study of Parents and Children (ALSPAC), an ongoing UK population-based study. The study website contains details of all data that is available through a searchable data dictionary 〈http://www.bris.ac.uk/alspac/researchers/data-access/data-dictionary〉. Ethical approval for the study was obtained from the ALSPAC Ethics and Law Committee and the Local Research Ethics Committees. We restricted our sample to participants recruited during Phase I in order to include covariate information collected during early infancy (this data is not available for Phase II participants). During Phase I enrolment, 14,541 pregnant mothers residing in the former Avon Health Authority in the south-west of England with expected dates of delivery between 1 April 1991 and 31 December 1992 were recruited to the study. These pregnancies resulted in 14,062 live births, of which 13,617 singletons were alive at 1 year of age. For further details on the cohort profile, representativeness and phases of recruitment see Boyd et al. (2013).

### 2.2 Measures

#### 2.2.1 Exposure: socioeconomic adversity

**Fig. 1:**
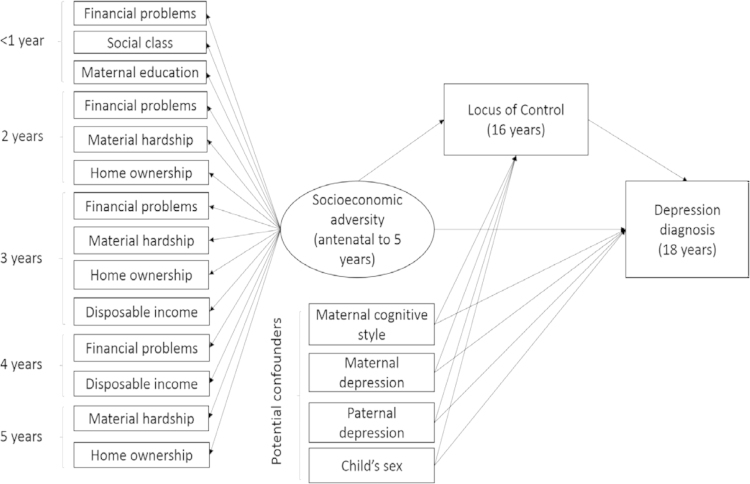
Measurement model of the hypothesised associations among socioeconomic adversity in early life, locus of control and depression diagnosis at 18 years, adjusted for potential confounders. *Note*: observed variables are represented by squares, whilst the latent variable is represented by circle. Covariances are not shown to reduce figure complexity.

We used 14 binary indicators derived from questionnaires administered to mothers in the antenatal period and during the first 5 years of the study child's life to derive a normally distributed latent factor of socioeconomic adversity (Fig. 1). The variables assessed in the antenatal period were: maternal educational attainment classified as none/minimal (mothers with the lowest level of qualifications generally obtained at age 16 years, vocational qualifications, or none) versus higher-level qualifications (mothers with ordinary-level qualifications generally obtained at age 16 years/advanced-level qualifications generally obtained at age 18 years/university degree); social class assessed on the basis of the lower of the mother's or partner's occupational social class using the 1991 British Office of Population and Census Statistics classification and dichotomised into social class I–IV (professional, managerial, or skilled professionals) and V–VI (partly skilled or unskilled occupations); and financial problems (occurrence of major financial problems versus none). The following socioeconomic adversity indicators were assessed repeatedly from birth to 5 years (Fig. 1): financial problems (yes/no); home ownership defined as living in owner–occupier or privately rented accommodation versus subsidised housing; material hardship derived using a cut-off of ≥5 corresponding to material hardship scores in the top 20% of the sample; and low family disposable income derived from a continuous weekly income measure and dichotomised to comprise those who were in the lowest income quartile versus the rest of the sample.

#### 2.2.2 Outcome: depression

Depression was assessed using the Clinical Interview Schedule-Revised (CIS-R; Lewis, 1994) at a research clinic attended at mean age 17.8 years (hereafter referred to as 18 years). Participants completed a self-administered computerised version of the CIS-R, which measures current symptoms across multiple domains. Computer algorithms were used to identify psychiatric disorders according to DSM-IV and ICD-10 diagnostic criteria (Lewis, 1994). The CIS-R is designed for, and has been widely used with community samples in the UK and elsewhere (e.g., Clark et al., 2007; Jenkins et al., 1997). Good agreement has been demonstrated between administration by a clinically trained interviewer, lay interviewer and self-administration using the computerised version (Lewis, 1994). Based on this interview we derived a binary variable to indicate presence versus absence of a depressive disorder.

#### 2.2.3 Mediator: locus of control

Adolescents completed a 12-item shortened version of the Nowicki–Strickland Internal–External scale (CNSIE; Nowicki, 1976; Nowicki and Duke, 1974; Nowicki and Strickland, 1973) (see Supplementary material) as a part of face-to-face clinic assessment at age 16 (median age at completion=16.7; inter-quartile range=16.6–16.10). A person with a higher ‘internal’ score on this measure is considered to perceive that the outcome of events is under their own control, whilst a person with a higher ‘external’ score on this measure is considered to perceive that the outcome of events is controlled by outside circumstances. A total score was derived by summing scores for all the items, with higher scores indicating a more external LoC. The questionnaire has been shown to have good construct validity and test–retest reliability in children from ages 9 through 18 years (Nowicki, 1976; Nowicki and Duke, 1974; Nowicki and Strickland, 1973) and has been used extensively in the previous research (Nowicki and Duke, 2013). For participants missing responses to one or two locus of control items (e.g., less than 20% of the total scale items), unanswered questions were replaced with the mean of the participants' own responses to the rest of the scale items.

#### 2.2.4 Confounding variables

Child's gender, maternal and paternal depressive symptoms and maternal cognitive style were included as potential confounders as they have been previously shown to be associated with exposure to childhood adversity, locus of control and depression (Edwards et al., 2003; Klein et al., 2005; Paulson et al., 2006; Solantaus et al., 2004). Maternal and paternal self-reported depression was assessed using the Edinburgh Postnatal Depression Scale (EPDS; Cox et al., 1987) administered when the study of child was 8 months. Maternal cognitive style was assessed using a 6-item scale (Evans et al., 2005) derived from a broader measure of intrapersonal sensitivity (Boyce and Parker, 1989) and administered at 18 weeks gestation. The six items comprising the scale map onto negative cognitions outlined in Beck's cognitive theory of depression (e.g., ‘I always expect criticism’ see Evans et al. (2005)). The scores from the six items were summed up to derive a total negative cognitive style score (range=0–18), with higher scores reflecting more negative cognitions.

### 2.3 Statistical analyses

Descriptive statistics were obtained using STATA 12.0. Primary analyses were conducted using Mplus software version 7.11 (Muthén and Muthén, 2012) using the WLSMV estimator. Prior to examining the hypothesised mediation pathway, we first tested a measurement model incorporating the outcome (binary variable indicating diagnosis of depression), exposure (latent factor of early socioeconomic adversity), hypothesised mediator (continuous locus of control score) and all potential confounders (child's sex, maternal cognitive style, maternal and paternal depression). The measurement model is illustrated in Fig. 1. Early socioeconomic adversity was estimated as a latent variable comprising the 14 binary indicators of socioeconomic disadvantage described above. Residual variances of the repeated early adversity indicators were allowed to co-vary to accommodate common method variance at each assessment. The exposure variable and potential confounders were also free to co-vary. Acceptability of the model fit was evaluated using standard goodness of fit indices. The chi-square test of exact fit is stringent and sensitive to sample size with simulations showing the test will routinely reject good models when sample size is large (e.g., *n*>200; Brown, 2006; Schumacker and Lomax, 2010), thus we considered several relative fit indices. A root mean square error of approximation (RMSEA) value less than 0.06, Tucker–Lewis index (TLI) and comparative fit index (CFI) values greater than 0.95 are considered indicative of good fit to the data (Hu and Bentler, 1999).

Once the measurement model had been confirmed, we tested a structural model to estimate the direct and indirect pathways of interest. As techniques to assess mediation progress, methodologists have emphasised the importance of considering the potential impact of mediator–outcome confounders within mediation models (e.g., Imai et al., 2010). Thus, we adopted the mediation approach recommended by Muthén (2011), which allows for the assessment of mediation effects within the context of potential mediator–outcome confounders. Using the “Model Constraint” command, new parameters and standard errors representing causally-defined direct and indirect effects (Robins and Greenland, 1992; Valeri and VanderWeele, 2013) were calculated from model estimated parameters. For a detailed description and Mplus input syntax see Muthén (2011). First we estimated an unadjusted mediation model that included only the exposure (early socioeconomic adversity), mediator (locus of control at 16 years) and outcome (diagnosed depression at 18 years). Next we estimated the model adjusted for the child's sex. The final model was adjusted for the child's sex and potential maternal and paternal confounders. We calculated bootstrapped standard errors and confidence intervals from 1000 bootstrap samples (MacKinnon et al., 2004) to account for non-normality associated with a binary outcome.

### 2.4 Missing data

Complete-case analyses can be biased if data are not missing completely at random. In order to examine the impact of response attrition on our conclusions, we examined characteristics of the complete-case sample compared with the rest of the ALSPAC cohort. We used STATA 12.0 to impute 50 datasets, each entailing 20 cycles of regression switching, using multiple imputation by chained equations (Royston, 2009). This is a recommended procedure for missing data (Sterne et al., 2009) which assumes data are missing at random (MAR) conditional on the variables in the imputation model. Our imputation model included a number of auxiliary socio-demographic and mental health variables predictive of incomplete variables and/or missingness, including locus of control score at 8 years and depressive symptoms scores from ages 10 through 19 years. In order to ensure plausibility of the MAR assumption, cases were included in the imputation sample only if data were available for depressive symptoms and each socioeconomic adversity indicator on at least one measurement occasion. Therefore we imputed data for a sample of 6851 participants. Predictive mean matching was employed for non-normal variables (White et al., 2011). The imputed data was imported into Mplus and mediation analyses were repeated over the 50 imputed datasets combining the estimates according to Rubin's rules (Royston et al., 2009).

## 3 Results

### 3.1 Descriptive characteristics

The starting sample was 8803 participants for whom data were available on each socioeconomic adversity indicator at one or more time points. Of these, 3528 participants (40.1%) completed the CIS-R diagnostic interview at 18 years, and 4074 participants (46.3%) completed the locus of control measure at 16 years. Complete data for the exposure (socioeconomic adversity), outcome (diagnosed depression) and mediator (locus of control) were available for 2663 participants. For 1892 of these participants, complete data were also available for all potential confounders. Given the considerable response attrition, sensitivity analyses on multiply imputed data examined the impact of attrition on our conclusions. Descriptive characteristics for the complete case sample compared to partial responders are provided in Table 1. Participants with complete data came from more socially advantaged families with fewer depressive symptoms.

### 3.2 Measurement model

The measurement model incorporating early adversity, locus of control and potential confounders is shown in Fig. 1. Fit statistics indicated that the measurement model fit the data well (RMSEA=0.04, 95% CI: 0.03–0.04; TFI=0.97; CFI=0.97). This supported the adequacy of the model for subsequent tests of structural paths and mediation.

### 3.3 Association among early socioeconomic adversity, locus of control and depression

Prior to examining the hypothesised mediation pathway, we examined the univariable associations among socioeconomic adversity, LoC and depression. There was evidence that greater early socioeconomic adversity was associated with an increased risk of depression at 18 years (*β*=0.191; 95% BC CI: 0.055–0.340, *p*=0.007). There was also evidence that more external locus of control at 16 years was associated with increased risk of diagnosed depression at 18 years (*β*=0.105; 95% BC CI: 0.069–0.136, *p*<0.001). In order to assist interpretation of the size of these probit estimates, Table 2 shows the predicted probability of depression diagnosis at different levels (±1 and 2 standard deviations) of early socioeconomic adversity and locus of control. Experiences of socioeconomic adversity were associated with LoC orientation (*β*=0.752; 95% BC CI: 0.583–0.973, *p*<0.001).

### 3.4 Mediation model

A series of models were estimated to assess the hypothesised mediation pathway. Table 3 shows parameter estimates, bootstrapped standard errors and bias-corrected (BC) confidence intervals for the unadjusted and adjusted models. Within the unadjusted model, there was strong evidence of an indirect pathway from early social adversity to diagnosed depression at 18 years via locus of control (*β*=0.123; 95% BC CI: 0.073–0.185, *p*<0.001). There was weak evidence of a direct pathway from early social adversity to diagnosed depression once the indirect effect via locus of control was accounted for (*β*=0.216; 95% BC CI: −0.008 to 0.484, *p*=0.088).

**Fig. 2:**
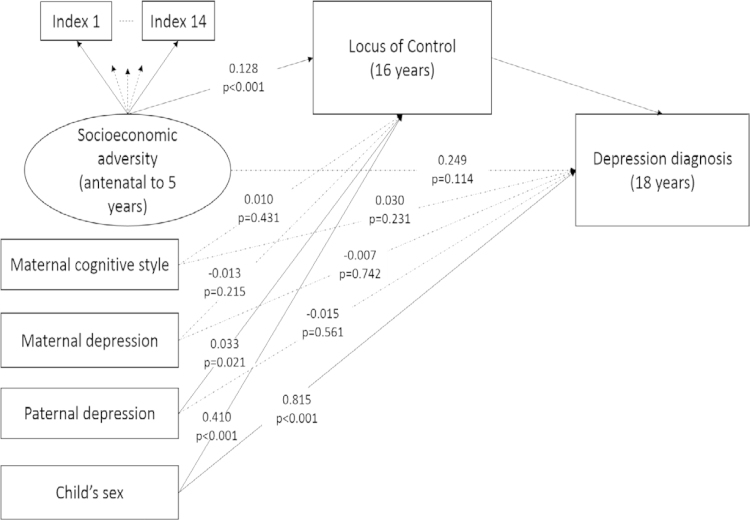
Structural mediation model estimating the direct and indirect pathways from early social adversity to diagnosed depression at 18 years, adjusted for potential confounders (*n*=2663). *Note*: Path coefficients on the edges are unstandardised regression estimates. Pathways delineated as dash lines are statistically non-significant (*p*>0.05).

Adjustment for child's sex (adjusted model 1) and maternal and paternal characteristics (adjusted model 2) made little difference to the parameter estimates. Within the fully adjusted model, there remained strong evidence of an indirect path from early social adversity through locus of control to diagnosed depression at 18 years (*β*=0.128; 95% BC CI: 0.073–0.195, *p*<0.001). This indirect path via locus of control accounted for 34% of the total estimated association between early socioeconomic adversity and diagnosed depression. Path estimates for the fully adjusted mediation model are illustrated in Fig. 2. There was strong evidence that child's sex was also associated with diagnosed depression (*β*=0.815; 95% BC CI: 0.516–1.156, *p*<0.001), as well as locus of control at 16 years (*β*=0.410; 95% BC CI: 0.255–0.549, *p*<0.001). The direction of these associations indicates that females were more likely to be diagnosed with depression and report more external locus of control. There was also some evidence to suggest that offspring of fathers with higher depression reported more external locus of control at 16 years (*β*=0.033; 95% BC CI: 0.004–0.061, *p*=0.021).

### 3.5 Missing data: sensitivity analyses

In order to examine the impact of response attrition on our findings, mediation analyses were repeated using 50 imputed datasets for a sample of 6851 participants. Results from these analyses are presented in Table 4. The resulting fraction of missing information (FMI) estimates (Schafer, 1997) indicated that 50 imputed datasets were sufficient. The results from analyses with imputed data supported our findings: the direct and indirect effect estimates were in the same direction and led to the same overarching conclusions. However, the sizes of the observed direct and indirect effects were greater in the imputed data. Although it is not possible to entirely account for the impact of response attrition, the pattern of missing data and analyses suggest that attrition lead to an underestimation of the direct and indirect effects size in the complete case analysis. This was most apparent for the remaining direct effect from early socioeconomic adversity to diagnosed depression. Within the fully adjusted analysis using imputed data, the indirect path from early socioeconomic adversity to diagnosed depression through locus of control was estimated as *β*=0.192 (*p<*0.001), while the remaining direct pathway from early socioeconomic adversity to depression was estimated as *β*=0.522 (*p=*0.002). Based on analyses using imputed data, we would estimate a slightly lower proportion (27%) of the total association between early socioeconomic adversity and depression is accounted for by the indirect path through locus of control.

## 4 Discussion

### 4.1 Main findings

We examined whether exposure to early socioeconomic adversity is associated with LoC in adolescence and a diagnosis of depression at 18 years. We further investigated whether LoC mediates the association between socioeconomic adversity and depression in young adulthood. We found evidence that exposure to early socioeconomic adversity is associated with more external LoC orientation at 16 years, which, in turn, is associated with depression at 18 years. This finding is consistent with previous research linking external LoC to depression (Harrow et al., 2009; Twenge et al., 2004) and highlights important contributions of perceived sense of control in development of depression. Approximately 34% (27% in analyses with imputed data) of the total estimated association between socioeconomic adversity and diagnosed depression was accounted for by the indirect path through external LoC in the model adjusted for child's gender and various parental characteristics. This finding is consistent with studies supporting the mediating role of LoC in the association between exposure to early adversity and depression in young adulthood (Deardorff et al., 2003; Hunter et al., 2010; Kliewer and Sandler, 1992). Although attenuated, there was evidence of a direct pathway from early socioeconomic adversity to depression once the indirect effect via locus of control was accounted for, suggesting an independent effect of early adversity on development of depression.

### 4.2 Strength and limitations

The current study has several strengths, including a longitudinal design, a large community-based sample, a measure of clinical diagnosis of depression as an outcome, and adjustment for a range of confounders. To our knowledge, no previous prospective longitudinal study has examined LoC orientation as a mechanism of the association between early socioeconomic adversity and depression in young adulthood. Modelling early socioeconomic adversity as a latent variable enabled us to capture exposure to various indices of socioeconomic adversity from birth to 5 years. A limitation of the study relates to sample attrition, which is strongly associated with socioeconomic disadvantage in the ALSPAC and this has important implications for the internal validity of the study. In particular, participants from lower socioeconomic background and those with mental health problems were underrepresented in our sample. However, the attrition rates in this cohort are similar to those observed in other large-scale longitudinal studies (Callaway et al., 2007). The pattern of missing data and results of the sensitivity analyses suggest that attrition lead to an underestimation of the direct and indirect effect sizes in the complete case analysis. Repeating the analyses with the imputed sample adjusted for the bias introduced by missing data and improved efficiency compared to complete case analysis (Klebanoff and Cole, 2008; Spratt et al., 2010).

### 4.3 Alternative mechanisms

A proportion of the association between early socioeconomic adversity and depression was not explained by external LoC orientation. This finding is in line with previous studies examining LoC as a pathway between childhood adversity and mental health problems (Fisher et al., 2013). This could indicate direct traumatic effect of exposure to childhood adversity on subsequent development of depression in young adulthood via biological systems such as the hypothalamic pituitary axis (HPA; Penza et al., 2003). Indeed, accumulating evidence suggests that childhood adversity is associated with HPA dysregulation and heightened stress reactivity in adolescents and adults (Heim et al., 2000; McLaughlin et al., 2009), which, in turn, may lead to maladaptive emotional and social functioning. Similarly, there is evidence to suggest that individuals with external LoC tend to display heightened neuroendocrine and autonomic stress responsiveness (Declerck et al., 2006; Steptoe and Willemsen, 2004), whereas individuals with internal LoC show lower cortisol responses to stress (Pruessner et al., 1997). Although there is no longitudinal research to support this assumption, it could be that dysregulation of HPA axis is a common neurobiological mechanism linking early life adversity, development of maladaptive control-related beliefs and depression.

The unexplained proportion of the association between socioeconomic adversity and depression via external LoC could also be due to other mediating factors or residual confounding not accounted for in the present analyses. It has been suggested that the link between early life adversity and negative mental health outcomes in adulthood, including depression, could be explained by low self-esteem, interpersonal difficulties, and maladaptive coping strategies (Whiffen and MacIntosh, 2005). In addition, experiences of poverty and material hardship in childhood often co-occur with emotional and physical neglect, abuse and victimisation, which are, in turn, strong predictors of adolescent and adulthood depression (Turner et al., 2006). Our assessment of early life adversity did not include questions on more severe forms such as abuse and other trauma, thus, possible mediating effect of these factors could not be examined in this study. In addition, individual sense of control is believed to be a complex, multi-dimensional construct better conceptualised as a combination of LoC, self-efficacy, learned helplessness, and an individual's desire of control (Shapiro et al., 1993). Examination of this multidimensional concept of control beliefs was beyond the scope of our study.

Mechanisms that could explain the link between experiences of early socioeconomic adversity and external LoC also warrant further examination. It has been suggested that early experiences of poverty may foster external LoC orientation in children through exposure to parental depression and negative parenting (Chorpita, 2001; Gilman et al., 2003). Indeed, there is some longitudinal evidence to support a link between negative parenting practises and external LoC orientation (Muris et al., 2004), whilst parental warmth has been linked to a more internal LoC (Carton and Nowicki, 1994). Additional studies which test complex mediational models are warranted to provide further insights into multiple pathways among early socioeconomic adversity, LoC and depression.

Although the study controlled for a range of prospectively measured parental and child characteristics, we did not examine possible genetic confounders that may explain observed associations. Genetic influences explain approximately 40–50% of the variance in depression (Levinson, 2006), however, there is little evidence on the heritability of LoC. The few studies that examined genetic influences on individual differences in LoC estimate these to be between 10% and 55% (Johansson et al., 2001). It is, therefore, possible that the association between external LoC and depression may be partly explained by common genes that contribute to both. Results from future genetically informative designs may provide further insights towards understanding of the mechanisms underlying this association.

### 4.4 Other associations of interest

Other associations of interest emerged in the context of the present study. Consistent with numerous epidemiological studies girls in our sample were more likely to meet criteria for a depression diagnosis (Parker and Brotchie, 2010) and reported more external LoC (Feingold, 1994) than boys. Gender differences in depression are well-documented with differences in cognitive functioning and more frequent exposure to adverse experiences in childhood often cited as contributing factors (Piccinelli and Wilkinson, 2000). There is also some longitudinal evidence to suggest that girls move toward more external LoC disposition during middle adolescence, whilst boys become more internal (Kulas, 1996; Ross and Mirowsky, 2002). However, these findings are inconsistent and require further longitudinal research for adequate replication. Interestingly, the findings of this study suggest more external LoC orientation in offspring of depressed fathers. Research on parental depression supports the importance of studying fathers in relation to child outcomes (Ramchandani and Psychogiou, 2009). Paternal depression is associated with more parent–child conflict (Kane and Garber, 2004) and harsh disciplining (Schacht et al., 2009), which, in turn, has been linked to more external LoC in children and adolescents (Lynch et al., 2002). Although mother–child conflict may be more frequent than father–child conflict, it has been suggested that the latter may be more harmful to children's behavioural and emotional development (Forehand et al., 1987). Thus, the strong influence of paternal depression and associated coercive parenting could explain this finding.

### 4.5 Clinical implications

The findings of the present study have important implications for depression prevention programs. Although LoC is thought to be a relatively enduring individual characteristic (Kulas, 1996), it has been suggested that it is amenable to psychological interventions, particularly in childhood and adolescence (deCharms, 1976; Trice, 1990). Evidence indicates that programs focusing on restructuring cognitive coping strategies and control-related beliefs result in shifts in LoC from less external to more internal orientation (Figurelli and Hartman, 1994). Internal LoC, in turn, is associated with better adherence to treatment (Steel et al., 2000) and favourable therapy outcomes (Delsignore and Schnyder, 2007; Weisz, 1986). Our findings suggest that depression prevention programs should include a component that addresses cognitive beliefs about control because shifting external LoC orientation to internal could help to reduce the risk of developing depression.

## Role of funding source

The UK Medical Research Council, the Wellcome Trust (Grant ref.: 102215/2/13/2) and the University of Bristol provide core support for ALSPAC.

## Conflict of interest

All authors declare that they have no conflicts of interest.

## Figures and Tables

**Table 1:** Individual and family characteristics for the complete sample and partial responders.

	Initial ALSPAC sample (*n*=13,617)					
	Complete case (*n*=2663)		Partial data (*n*=10,954)		Statistical test^a^	
Categorical measures	(%)		(%)		*χ*^2^	
Female	58.2		46.0		*χ*^2^(1)=128.0⁎⁎	
Maternal education						
*Degree*	22.9		10.0		*χ*^2^(3)=610.4**	
*Advanced high school*	29.3		20.6			
*Standard high school*	33.2		35.0			
*No high school*	14.7		34.4			
Parental social class						
I *Professional*	20.8		11.0		*χ*^2^(4)=322.7⁎⁎	
II *Managerial/technical*	46.6		40.2			
III *Skilled non-manual*	21.9		26.7			
IV *Skilled manual*	7.6		15.2			
IV & V *Partly or unskilled*	3.0		6.8			
Low family income						
*Child age 3 years*	11.2		22.5		*χ*^2^(1)=144.4⁎⁎	
*Child age 4 years*	14.8		28.9		*χ*^2^(1)=186.6⁎⁎	
Does not own home						
*Child age 8 months*	10.3		26.9		*χ*^2^(1)=307.5⁎⁎	
*Child age 2 years*	10.7		25.4		*χ*^2^(1)=243.6⁎⁎	
*Child age 3 years*	10.6		24.1		*χ*^2^(1)=207.6⁎⁎	
*Child age 5 years*	9.8		22.7		*χ*^2^(1)=193.9**	
Major financial problems						
*Child age 8 months*	11.9		15.7		*χ*^2^(1)=22.2⁎⁎	
*Child age 2 years*	12.2		15.8		*χ*^2^(1)=20.2⁎⁎	
*Child age 3 years*	12.9		16.5		*χ*^2^(1)=18.2⁎⁎	
*Child age 4 years*	10.3		13.4		*χ*^2^(1)=16.3⁎⁎	
Material hardship						
*Child age 8 months*	22.6		31.2		*χ*^2^(1)=71.5⁎⁎	
*Child age 2 years*	20.2		29.7		*χ*^2^(1)=86.8⁎⁎	
*Child age 3 years*	19.7		27.4		*χ*^2^(1)=59.9⁎⁎	
*Child age 4 years*	15.0		20.9		*χ*^2^(1)=40.9⁎⁎	
Offspring depression diagnosis	7.1		9.1		*χ*^2^(1)=5.5⁎⁎	
18 years						

Continuous measures	Mean	SD	Mean	SD	Mean diff	95% CI

Offspring locus of control	3.0	(2.0)	3.4	(2.2)	0.42	0.30–0.54
16 years						
Maternal cognitive style	4.9	(3.4)	5.0	(3.7)	0.13	−0.3 to 0.28
Maternal depression	4.9	(4.4)	5.6	(4.8)	0.67	0.46–0.87
Paternal depression	3.2	(3.5)	3.4	(3.8)	0.22	0.02–0.41

*Note*: ALSPAC Avon Longitudinal Study of Parents and Children.

a Differences in sample characteristics according to response attritions were tested using chi-square tests for categorical variables and *t* tests for continuous variables.

^⁎⁎^ *p*<0.001.

**Table 2:** Estimated prevalence of CIS-R depression diagnosis at varying levels of early social adversity and locus of control.

	Estimated prevalence (%) of depression				
Predictor variable	−2 SDs	−1 SDs	Mean	+1 SDs	+2 SDs
Socioeconomic adversity	4.1	5.3	6.9	8.9	11.2
(Latent factor)					
Locus of control	1.3	2.1	3.5	5.4	8.1
(Total score)					

*Note*: This table shows estimated prevalence of diagnosed depression at 1 and 2 standard deviations above and below the mean for each predictor variable. Estimates are derived from univariable model with no other confounders included. CIS-R Clinical Interview Schedule-Revised.

**Table 3:** Estimates using complete case data (*n*=2663)^a^ of the direct and indirect effects of early socioeconomic adversity on depression diagnosis at 18 years mediated through locus of control at 16 years.

	Model estimates			
	*β*	SE	*p*	BC 95% CI^b^
*Unadjusted model*				
1. *Total effect*	0.339	0.124	0.006	0.098–0.598
Early adversity on depression diagnosis at 18				
2. *Indirect effect*	0.123	0.027	<0.001	0.073–0.185
Early adversity on depression diagnosis at 18, through locus of control at 16				
3. *Remaining direct effect*	0.216	0.127	0.088	−0.008 to 0.484
Early adversity on depression diagnosis at 18, adjusted for locus of control				

*Adjusted* 1 (*gender*)				
1. *Total effect*	0.386	0.152	0.011	0.096–0.692
Early adversity on depression diagnosis at 18				
2. *Indirect effect*	0.135	0.032	<0.001	0.074–0.204
Early adversity on depression diagnosis at 18, through locus of control at 16				
3. *Remaining direct effect*	0.251	0.155	0.104	−0.023 to 0.577
Early adversity on depression diagnosis at 18, adjusted for locus of control				

*Adjusted* 2 (*gender, maternal & paternal factors*)				
1. *Total effect*	0.377	0.156	0.016	0.074–0.675
Early adversity on depression diagnosis at 18				
2. *Indirect effect*	0.128	0.030	<0.001	0.073–0.195
Early adversity on depression diagnosis at 18, through locus of control at 16				
3. *Remaining direct effect*	0.249	0.158	0.114	−0.032 to 0.574
Early adversity on depression diagnosis at 18, adjusted for locus of control				

a Analyses restricted to participants with complete mediator (locus of control) and outcome (depression diagnosis) data.

b BC 95% CI: bias corrected (1000 bootstrap samples).

**Table 4:** Estimates using imputed data (*n*=6851) of the direct and indirect effects of early socioeconomic adversity on depression diagnosis at 18 years mediated through locus of control at 16 years.

	Model estimates			
	*β*	SE	*p*	FMI^a^
*Unadjusted model*				
1. *Total effect*	0.584	0.120	<0.001	0.330
Early adversity on depression diagnosis at 18				
2. *Indirect effect*	0.152	0.032	<0.001	0.389
Early adversity on depression diagnosis at 18, through locus of control at 16				
3. *Remaining direct effect*	0.433	0.124	<0.001	0.337
Early adversity on depression diagnosis at 18, adjusted for locus of control				

*Adjusted* 1 (*gender*)				
1. *Total effect*	0.581	0.121	<0.001	0.328
Early adversity on depression diagnosis at 18				
2. *Indirect effect*	0.149	0.032	<0.001	0.390
Early adversity on depression diagnosis at 18, through locus of control at 16				
3. *Remaining direct effect*	0.432	0.125	0.001	0.336
Early adversity on depression diagnosis at 18, adjusted for locus of control				

*Adjusted* 2 (*gender, maternal & paternal factors*)				
1. *Total effect*	0.714	0.166	<0.001	0.429
Early adversity on depression diagnosis at 18				
2. *Indirect effect*	0.192	0.042	<0.001	0.389
Early adversity on depression diagnosis at 18, through locus of control at 16				
3. *Remaining direct effect*	0.522	0.172	0.002	0.342
Early adversity on depression diagnosis at 18, adjusted for locus of control				

a FMI: Fraction of Missing Information.

## Footnotes

Supplementary data associated with this article can be found in the online version at doi:10.1016/j.jad.2015.05.030.

## References

[bib1] Barrera M., Prelow H.M., Dumka L.E., Gonzales N.A., Knight G.P., Michaels M.L., Roosa M.W., Tein J. (2002). Pathways from family economic conditions to adolescents' distress: supportive parenting, stressors outside the family, and deviant peers. J. Community Psychol..

[bib2] Benassi V.A., Dufour C.L., Sweeney P.D. (1988). Is there a relationship between locus of control orientation and depression?. J. Abnorm. Psychol..

[bib3] Bosma H., van de Mheen H.D., Mackenbach J.P. (1999). Social class in childhood and general health in adulthood: questionnaire study of contribution of psychological attributes. Br. Med. J..

[bib4] Boyce P., Parker G. (1989). Development of a scale to measure interpersonal sensitivity. Aust. N. Z. J. Psychiatry.

[bib5] Boyd A., Golding J., Macleod J., Lawlor D.A., Fraser A., Henderson J., Molloy L., Ness A., Ring S., Smith G.D. (2013). Cohort profile: the ‘children of the 90s'—the index offspring of the Avon Longitudinal Study of Parents and Children. Int. J. Epidemiol..

[bib6] Brown T.A. (2006). Confirmatory Factor Analysis for Applied Research.

[bib7] Bryant B.K., Trockel J.F. (1976). Personal history for psychological stress related to locus of control orientation among college women. J. Consult. Clin. Psychol..

[bib8] Callaway L.K., McIntyre H.D., O'Callaghan M., Williams G.M., Najman J.M., Lawlor D.A. (2007). The association of hypertensive disorders of pregnancy with weight gain over the subsequent 21 years: findings from a prospective cohort study. Am. J. Epidemiol..

[bib9] Carton J.S., Nowicki S. (1996). Origins of generalized control expectancies: reported child stress and observed maternal control and warmth. J. Soc. Psychol..

[bib10] Carton J.S., Nowicki S. (1994). Antecedents of individual differences in locus of control of reinforcement. Genet. Soc. Gen. Psychol. Monogr..

[bib11] Chapman D.P., Whitfield C.L., Felitti V.J., Dube S.R., Edwards V.J., Anda R.F. (2004). Adverse childhood experiences and the risk of depressive disorders in adulthood. J. Affect. Disord..

[bib12] Chorpita B.F., Vasey M.W., Dadds M.R. (2001). Control and development of negative emotion. The Developmental Psychopathology of Anxiety.

[bib13] Chorpita B.F., Barlow D.H. (1998). The development of anxiety: the role of control in the early environment. Psychol. Bull..

[bib14] Clark C., Rodgers B., Caldwell T., Power C., Stansfeld S. (2007). Childhood and adulthood psychological ill health as predictors of midlife affective and anxiety disorders: the 1958 British birth cohort. Arch. Gen. Psychiatry.

[bib15] Cole D.A., Jacquez F.M., Maschman T.L. (2001). Social origins of depressive cognitions: a longitudinal study of self-perceived competence in children. Cogn. Ther. Res..

[bib16] Conger R.D., Wallace L.E., Sun Y., Simons R.L., McLoyd V.C., Brody G.H. (2002). Economic pressures in African American families: a replication and extension of the family stress model. Dev. Psychol..

[bib17] Cox J., Holden J., Sagovsky R. (1987). Detection of postnatal depression. Development of the 10-item Edinburgh Postnatal Depression Scale. Br. J. Psychiatry.

[bib18] Deardorff J., Gonzales N.A., Sandler I.N. (2003). Control beliefs as a mediator of the relation between stress and depressive symptoms among inner-city adolescents. J. Abnorm. Child Psychol..

[bib19] deCharms R. (1976). Enhancing Motivation.

[bib20] Declerck C.H., Boone C., De Brabander B. (2006). On feeling in control: a biological theory for individual differences in control perception. Brain Cognit..

[bib21] Delsignore A., Schnyder U. (2007). Control expectancies as predictors of psychotherapy outcome: a systematic review. Br. J. Clin. Psychol..

[bib22] Dong M., Anda R.F., Felitti V.J., Dube S.R., Williamson D.F., Thompson T.J., Loo C.M., Giles W.H. (2004). The interrelatedness of multiple forms of childhood abuse, neglect, and household dysfunction. Child Abuse Negl..

[bib23] Donnelly M. (1999). Factors associated with depressed mood among adolescents in Northern Ireland. J. Community Appl. Soc. Psychol..

[bib24] Edwards V.J., Holden G.W., Felitti V.J., Anda R.F. (2003). Relationship between multiple forms of childhood maltreatment and adult mental health in community respondents: results from the adverse childhood experiences study. Am. J. Psychiatry.

[bib25] Evans J., Heron J., Lewis G., Araya R., Wolke D. (2005). Negative self-schemas and the onset of depression in women: longitudinal study. Br. J. Psychiatry.

[bib26] Feingold A. (1994). Gender differences in personality: a meta-analysis. Psychol. Bull..

[bib27] Figurelli G.A., Hartman B.W. (1994). Assessment of change in scores on personal control orientation and use of drugs and alcohol of adolescents who participate in a cognitively oriented pretreatment intervention. Psychol. Rep..

[bib28] Fisher H.L., Schreier A., Zammit S., Maughan B., Munafò M.R., Lewis G., Wolke D. (2013). Pathways between childhood victimization and psychosis-like symptoms in the ALSPAC birth cohort. Schizophr. Bull..

[bib29] Forehand R., McCombs A., Brody G.H. (1987). The relationship between parental depressive mood states and child functioning. Adv. Behav. Res. Ther..

[bib30] Frenkel E., Kugelmass S., Nathan M., Ingraham L.J. (1995). Locus of control and mental health in adolescence and adulthood. Schizophr. Bull..

[bib31] Gilman S.E., Kawachi I., Fitzmaurice G.M., Buka S.L. (2003). Socio-economic status, family disruption and residential stability in childhood: relation to onset, recurrence and remission of major depression. Psychol. Med..

[bib32] Gilman S.E., Kawachi I., Fitzmaurice G.M., Buka S.L. (2002). Socioeconomic status in childhood and the lifetime risk of major depression. Int. J. Epidemiol..

[bib33] Grant K.E., Compas B.E., Thurm A.E., McMahon S.D., Gipson P.Y., Campbell A.J., Krochock K., Westerholm R.I. (2006). Stressors and child and adolescent psychopathology: evidence of moderating and mediating effects. Clin. Psychol. Rev..

[bib34] Grant K.E., Compas B.E., Stuhlmacher A.F., Thurm A.E., McMahon S.D., Halpert J.A. (2003). Stressors and child and adolescent psychopathology: moving from markers to mechanisms of risk. Schizophr. Bull..

[bib35] Haine R.A., Ayers T.S., Sandler I.N., Wolchik S.A., Weyer J.L. (2003). Locus of control and self-esteem as stress-moderators or stress-mediators in parentally bereaved children. Death Stud..

[bib36] Hammen C. (2005). Stress and depression. Annu. Rev. Clin. Psychol..

[bib37] Harrow M., Hansford B.G., Astrachan-Fletcher E.B. (2009). Locus of control: relation to schizophrenia, to recovery, and to depression and psychosis: a 15-year longitudinal study. Psychiatry Res..

[bib38] Heim C., Newport D.J., Heit S., Graham Y.P., Wilcox M., Bonsall R., Miller A.H., Nemeroff C.B. (2000). Pituitary-adrenal and autonomic responses to stress in women after sexual and physical abuse in childhood. J. Am. Med. Assoc..

[bib39] Hu L.T., Bentler P.M. (1999). Cutoff criteria for fit indexes in covariance structure analysis: conventional criteria versus new alternatives. Struct. Equ. Model..

[bib40] Hunter S.C., Durkin K., Heim D., Howe C., Bergin D. (2010). Psychosocial mediators and moderators of the effect of peer‐victimization upon depressive symptomatology. J. Child Psychol. Psychiatry.

[bib41] Imai K., Keele L., Yamamoto T. (2010). Identification, inference and sensitivity analysis for causal mediation effects. Stat. Sci..

[bib42] Jenkins R., Lewis G., Bebbington P., Brugha T., Farrell M., Gill B., Meltzer H. (1997). The National Psychiatric Morbidity surveys of Great Britain: initial findings from the household survey. Psychol. Med..

[bib43] Johansson B., Grant J.D., Plomin R., Pedersen N.L., Ahern F., Berg S., McClearn G.E. (2001). Health locus of control in late life: a study of genetic and environmental influences in twins aged 80 years and older. Health Psychol..

[bib44] Joinson, C., Kounali, D., Lewis, G.. A Prospective Cohort Study of Family Socioeconomic Position at Birth and Onset of Depressive Symptoms and Depression (unpublished results). 10.1007/s00127-016-1308-2 PMC5226994 27837235

[bib45] Kane P., Garber J. (2004). The relations among depression in fathers, children's psychopathology, and father–child conflict: a meta-analysis. Clin. Psychol. Rev..

[bib46] Kessler R.C., McLaughlin K.A., Green J.G., Gruber M.J., Sampson N.A., Zaslavsky A.M. (2010). Childhood adversities and adult psychopathology in the WHO World Mental Health Surveys. Br. J. Psychiatry.

[bib47] Kim L.S., Sandler I.N., Tein J.Y. (1997). Locus of control as a stress moderator and mediator in children of divorce. J. Abnorm. Child Psychol..

[bib48] Klebanoff M.A., Cole S.R. (2008). Use of multiple imputation in the epidemiologic literature. Am. J. Epidemiol..

[bib49] Klein D.N., Lewinsohn P.M., Rohde P., Seeley J.R., Olino T.M. (2005). Psychopathology in the adolescent and young adult offspring of a community sample of mothers and fathers with major depression. Psychol. Med..

[bib50] Kliewer W., Sandler I.N. (1992). Locus of control and self-esteem as moderators of stressor-symptom relations in children and adolescents. J. Abnorm. Child Psychol..

[bib51] Kulas H. (1996). Locus of control in adolescence: a longitudinal study. Adolescence.

[bib52] Lachman M.E., Weaver S.L. (1998). The sense of control as a moderator of social class differences in health and well-being. J. Pers. Soc. Psychol..

[bib53] Levinson D.F. (2006). The genetics of depression: a review. Biol. Psychiatry.

[bib54] Lewis G. (1994). Assessing psychiatric disorder with a human interviewer or a computer. J. Epidemiol. Community Health.

[bib55] Lynch S., Hurford D.P., Cole A. (2002). Parental enabling attitudes and locus of control of at-risk and honors students. Adolescence.

[bib56] Lyons-Ruth K., Wolfe R., Lyubchik A., Steingard R., Halfon N., McLearn K.T. (2002). Depressive symptoms in parents of children under age 3: sociodemographic predictors, current correlates, and associated parenting behaviors. Child Rearing in America: Challenges Facing Parents With Young Children.

[bib57] MacKinnon D.P., Lockwood C.M., Williams J. (2004). Confidence limits for the indirect effect: distribution of the product and resampling methods. Multivar. Behav. Res..

[bib58] Maughan B., Collishaw S., Argyris A. (2013). Depression in childhood and adolescence. J. Can. Acad. Child Adolesc. Psychiatry.

[bib59] McLaughlin K.A., Hatzenbuehler M.L., Hilt L.M. (2009). Emotion dysregulation as a mechanism linking peer victimization to internalizing symptoms in adolescents. J. Consult. Clin. Psychol..

[bib60] Muris P., Meesters C., Schouten E., Hoge E. (2004). Effects of perceived control on the relationship between perceived parental rearing behaviors and symptoms of anxiety and depression in nonclinical preadolescents. J. Youth Adolesc..

[bib61] Muthén, B., 2011. Applications of causally defined direct and indirect effects in mediation analysis using SEM in Mplus. Unpublished Working Paper, 〈www.statmodel.com〉.

[bib62] Muthén L.K., Muthén B.O. (2012). Mplus User's Guide.

[bib63] Nowicki S. (1976). Factor structure of locus of control in children. J. Genet. Psychol..

[bib64] Nowicki S., Duke M.P., Lefcourt H.M. (2013). The Nowicki–Strickland life-span locus of control scales: construct validation. Research With the Locus of Control Construct, Volume 2: Developments and Social Problems.

[bib65] Nowicki S., Duke M.P. (1974). A preschool and primary internal–external control scale. Dev. Psychol..

[bib66] Nowicki S., Strickland B. (1973). A locus of control scale for children. J. Consult. Clin. Psychol..

[bib67] Ostrander R., Herman K.C. (2006). Potential cognitive, parenting, and developmental mediators of the relationship between ADHD and depression. J. Consult. Clin. Psychol..

[bib68] Parker G., Brotchie H. (2010). Gender differences in depression. Int. Rev. Psychiatry.

[bib69] Paschall M.J., Hubbard M.L. (1998). Effects of neighbourhood and family stressors on African American male adolescents' self-worth and propensity for violent behavior. J. Consult. Clin. Psychol..

[bib70] Patten S.B., Wilkes T.C.R., Williams J.V.A., Lavorato D.H., El-Guebaly N., Schopflocher D. (2014). Retrospective and prospectively assessed childhood adversity in association with major depression, alcohol consumption and painful conditions. Epidemiol. Psychiatr. Sci..

[bib71] Paulson J.F., Dauber S., Leiferman J.A. (2006). Individual and combined effects of postpartum depression in mothers and fathers on parenting behavior. Pediatrics.

[bib72] Penza K.M., Heim C., Nemeroff C.B. (2003). Neurobiological effects of childhood abuse: implications for the pathophysiology of depression and anxiety. Arch. Women's Ment. Health.

[bib73] Piccinelli M., Wilkinson G. (2000). Gender differences in depression: critical review. Br. J. Psychiatry.

[bib74] Pruessner J.C., Gaab J., Hellhammer D.H., Lintz D., Schommer N., Kirschbaum C. (1997). Increasing correlations between personality traits and cortisol stress responses obtained by data aggregation. Psychoneuroendocrinology.

[bib75] Ramchandani P., Psychogiou L. (2009). Paternal psychiatric disorders and children's psychosocial development. Lancet.

[bib76] Robins J.M., Greenland S. (1992). Identifiability and exchangeability for direct and indirect effects. Epidemiology.

[bib77] Ross C.E., Mirowsky J. (2002). Age and the gender gap in the sense of personal control. Soc. Psychol. Q..

[bib78] Rotter J.B. (1966). Generalised expectancies for internal versus external control of reinforcement. Psychol. Monogr..

[bib79] Royston P. (2009). Multiple imputation of missing values: further update of ice, with an emphasis on categorical variables. Stata J..

[bib80] Royston P., Carlin J.B., White I.R. (2009). Multiple imputation of missing values: new features for mim. Stata J..

[bib81] Sandler I.N., Kim-Bae L.S., MacKinnon D. (2000). Coping and negative appraisal as mediators between control beliefs and psychological symptoms in children of divorce. J. Clin. Child Psychol..

[bib82] Schacht P.M., Cummings E.M., Davies P.T. (2009). Fathering in family context and child adjustment: a longitudinal analysis. J. Fam. Psychol..

[bib83] Schafer J.L. (1997). Analysis of Incomplete Multivariate Data.

[bib84] Shapiro D.H., Blinder B.J., Hagman J., Pituck S. (1993). A psychological “sense-of-control” profile of patients with anorexia nervosa and bulimia nervosa. Psychol. Rep..

[bib85] Schumacker R.E., Lomax R.G. (2010). A Beginner's Guide to Structural Equation Modeling.

[bib86] Solantaus T., Leinonen J., Punamäki R.L. (2004). Children's mental health in times of economic recession: replication and extension of the family economic stress model in Finland. Dev. Psychol..

[bib87] Spratt M., Carpenter J., Sterne J.A., Carlin J.B., Heron J., Henderson J., Tilling K. (2010). Strategies for multiple imputation in longitudinal studies. Am. J. Epidemiol..

[bib88] Stansfeld S.A., Clark C., Rodgers B., Caldwell T., Power C. (2011). Repeated exposure to socioeconomic disadvantage and health selection as life course pathways to mid-life depressive and anxiety disorders. Soc. Psychiatry Psychiatr. Epidemiol..

[bib89] Steel Z., Jones J., Adcock S., Clancy R., Bridgford‐West L., Austin J. (2000). Why the high rate of dropout from individualized cognitive‐behavior therapy for bulimia nervosa?. Int. J. Eat. Disord..

[bib90] Steptoe A., Willemsen G. (2004). The influence of low job control on ambulatory blood pressure and perceived stress over the working day in men and women from the Whitehall II cohort. J. Hypertens..

[bib91] Sterne J.A.C., White I.R., Carlin J.B., Spratt M., Royston P., Kenward M.G., Wood A.M., Carpenter J.R. (2009). Multiple imputation for missing data in epidemiological and clinical research: potential and pitfalls. Br. Med. J..

[bib92] Strickland B.R. (1989). Internal–external control expectancies: from contingency to creativity. Am. Psychol..

[bib93] Trice A.D. (1990). Adolescents' locus of control and compliance with contingency contracting and counselling interventions. Psychol. Rep..

[bib94] Twenge J.M., Zhang L., Im C. (2004). It's beyond my control: a cross-temporal meta-analysis of increasing externality in locus of control, 1960–2002. Pers. Soc. Psychol. Rev..

[bib95] Turner H.A., Finkelhor D., Ormrod R. (2006). The effect of lifetime victimization on the mental health of children and adolescents. Soc. Sci. Med..

[bib96] Valeri L., VanderWeele T.J. (2013). Mediation analysis allowing for exposure–mediator interactions and causal interpretation: theoretical assumptions and implementation with SAS and SPSS macros. Psychol. Methods.

[bib97] Weisz J.R. (1986). Contingency and control beliefs as predictors of psychotherapy outcomes among children and adolescents. J. Consult. Clin. Psychol..

[bib98] Whiffen V.E., MacIntosh H.B. (2005). Mediators of the link between childhood sexual abuse and emotional distress a critical review. Trauma Violence Abuse.

[bib99] White I.R., Royston P., Wood A.M. (2011). Multiple imputation using chained equations: issues and guidance for practice. Stat. Med..

