# Supplementary material for: Exposure to socioeconomic adversity in early life and risk of depression at 18 years: The mediating role of locus of control
Source: J Affect Disord. 2015 Sep 1;183:269–78. doi: 10.1016/j.jad.2015.05.030 (PMC4504028; doi:10.1016/j.jad.2015.05.030)
Supplement: Supplementary file 1 — Supplementary material [file mmc1.pdf]

Elsevier Editorial System(tm) for Journal of Affective Disorders
Manuscript Draft

Manuscript Number: JAD-D-15-00248R1

Title: Exposure to socioeconomic adversity in early life and risk of depression at 18 years: the mediating role of locus of control

Article Type: Research Paper

Keywords: Avon Longitudinal Study of Parents and Children (ALSPAC); early childhood; socioeconomic adversity; depression; locus of control.

Corresponding Author: Dr. Iryna Culpin, Ph.D.

Corresponding Author's Institution: University of Bristol, UK

First Author: Iryna Culpin, Ph.D.

Order of Authors: Iryna Culpin, Ph.D.; Lexine Stapinski, Ph.D.; Ömür B Miles, Ph.D.; Ricardo Araya , Ph.D. MRCPsych; Carol Joinson, Ph.D.

Abstract: Background: Previous studies have linked exposure to early socioeconomic adversity to depression, but the mechanisms of this association are not well understood. Locus of control (LoC), an individual's control-related beliefs, has been implicated as a possible mechanism, however, longitudinal evidence to support this is lacking.
Methods: The study sample comprised 8,803 participants from a UK cohort, the Avon Longitudinal Study of Parents and Children (ALSPAC). Indicators of early socioeconomic adversity were collected from the antenatal period to 5 years and modelled as a latent factor. Depression was assessed using the Clinical Interview Schedule-Revised (CIS-R) at 18 years. LoC was assessed with the Nowicki-Strickland Internal-External (CNSIE) scale at 16 years.
Results: Using structural equation modelling, we found that 34% of the total estimated association between early socioeconomic adversity and depression at 18 years was explained by external LoC at 16 years. There was weak evidence of a direct pathway from early socioeconomic adversity to depression after accounting for the indirect effect via external locus of control. Socioeconomic adversity was associated with more external LoC, which, in turn, was associated with depression.
Limitations: Attrition may have led to an underestimation of the direct and indirect effect sizes in the complete case analysis.
Conclusions: Results suggest that external LoC in adolescence is one of the factors mediating the link between early adversity and depression at 18 years. Cognitive interventions that seek to modify maladaptive control beliefs in adolescence may be effective in reducing risk of depression following early life adversity.

*Cover Letter

Iryna Culpin

School of Social and Community Medicine

University of Bristol

Oakfield House

Bristol

BS8 2BN

Email: iryna.culpin@bristol.ac.uk

Phone: (0117) 331 0162; Fax: (0117) 331 4026

11th May 2015

Professor C.L.E. Katona

Editor, *Journal of Affective Disorders*

Dear Professor Katona,

I have a pleasure in resubmitting the article entitled ‘Exposure to socioeconomic adversity in early life and risk of depression at 18 years: the mediating role of locus of control’ for your consideration as a paper to be published in the *Journal of Affective Disorders*. I, and my co-authors, confirm that the research reported in this article represents original material; have not been published anywhere else; and has been approved for resubmission by all co-authors.

The research described in this article is based on the sample from the Avon Longitudinal Study of Parents and Children (ALSPAC), an ongoing population-based study from South-West England (UK). In this study, we take advantage of the longitudinal data on various aspects of socioeconomic adversity measured in the antenatal period and through childhood (birth to 5 years), locus of control (LoC) at 16 years, and diagnosis of depression at 18 years to examine the role of LoC in the association between exposure to socioeconomic adversity and depression. I consider the submitted manuscript suitable for publication in the *Journal of Affective Disorders* because the subject matter addresses mechanisms linking early childhood

adverse experiences and subsequent psychopathology – a question that is of general interest to the readership.

The unique contribution of the present study is the longitudinal examination of LoC as a possible mechanism underlying the association between early life socioeconomic adversity and depression at 18 years. LoC has been theoretically implicated as a possible mechanisms, however, longitudinal evidence to support its mediating role is lacking with the majority of previous studies relying on cross-sectional assessment. In addition, we examine longitudinal associations between early life socioeconomic adversity and depression; socioeconomic adversity and LoC orientation; and LoC orientation and depression. We utilise a novel analytic approach recommended by Muthén (2011) which enables assessment of mediation effects within the context of potential mediator-outcome confounders using structural equation modelling (SEM).

In the revised version of the manuscript we address Reviewers' concerns with regard to the presentation issues and expand on the possible mechanisms that could explain the association between early socioeconomic adversity, LoC and depression. In addition, we address Reviewer #1 comments with regard to earlier measure of LoC and extra adjustments for childhood cognitive skills and depressive symptoms. As a corresponding author, I affirm that I have access to all data from the study and I take responsibility for the integrity of the data and accuracy of data analyses.

Yours Sincerely,

Iryna Culpin

Ms. Ref. No.: JAD-D-15-00248

Title: Exposure to socioeconomic adversity in early life and risk of depression at 18 years: the mediating role of locus of control

Comments from the Editor and Reviewers:

We thank the Editor and the Reviewers for their insightful comments. We have now revised the Manuscript in response to these comments and summarised the list of changes bellow.

Reviewer #1: This paper explores an important issue - possible mechanisms underlying the well-established association between exposure to early life adversity and later risk for depression. The analyses are based on the ALSPAC sample, a powerful longitudinal data-set for examining issues of this kind, and appear to have been well and appropriately conducted. The paper is clearly written, and reaches the interesting conclusion that external locus of control (LoC) in the mid-teens is likely to be one key mediator of the early-adversity—depression associations that are also found.

So what's not to like? Aside from the (hopefully minor) presentational issues noted below, my main concerns centred on the fact that only one model of the hypothesized associations was examined, whereas I suspect that others might obtain - or at least would be interesting to try and rule out. Three issues in particular struck me about the current formulation:

Comment:

1. Timing of the assessment of LoC: the main analyses use a measure of LoC taken at age 16 - but we learn from the Method section that there is also a measure at age 8. Why did the authors opt for the later measure - especially given that it is somewhat distal from the exposure of interest? Given that the authors note that LoC is often viewed as a relatively stable individual difference trait; I was puzzled why they had not either used the earlier measure, or perhaps derived an indicator of 'stably' external LoC from the two time-points.

Response:

The Reviewer is right to note that ALSPAC has a measure of LoC at 8 years. Although there is some evidence to suggest that LoC is a relatively enduring individual characteristic, the stability of the measure over time (8 years and 16 years) in the ALSPAC sample is relatively poor (r=0.218, n=3,801). This is in line with previous research on the stability of LoC indicating that control beliefs are still developing during childhood and begin to stabilise

during adolescence (Kulas, 1966). In addition, evidence suggests that as children age, they become more internal in their control beliefs (Skinner & Connell, 1986), and these differences in internality between younger children and adolescents may coincide with advances in cognitive development (Kliewer & Sandler, 1992). This is supported by ALSPAC data with younger children (8 years: M=5.97, SD=2.08) being more external than adolescents (16 years: M=3.18, SD=2.12). Although having a supportive and nurturing family environment has been linked to a more internal LoC (Carton & Nowicki, 1994), it may be that younger children are generally more external in their LoC orientation, given lack of control over events and circumstances, but gain more independence as they grow. Given that LoC may not be a truly time-invariant construct, with most changes in control tendencies still occurring among the young children, we have utilised LoC measure at 16 years as a more stable measure of individual's control beliefs most proximal to our outcome of interest, i.e. depression at 18 years.

Comment:

2. Reverse causation: there is of course ample justification from prior research for considering LoC as a risk factor for depression. It also seems possible, however, that prior experiences of depressed mood could increase the likelihood of maladaptive control beliefs - and as we learn (again from the Method section) that the study included repeated indicators of depressed mood from age 10 to age 19, I was surprised that this possibility had not been explored - or at the least that these prior measures had not been included as confounders.

Response:

We thank the Reviewer for the insightful comment. Our approach to choosing potential confounders was guided by the epidemiological literature that specify strict criteria for a variable to be a confounder: (1) it must be associated with the exposure; (2) it must be a cause of the outcome; (3) it should not be in the casual pathway between the potential risk factor and outcome (Rothman et al., 2008). The earliest time point at which childhood depressive symptoms were assessed is 10 years, which is after the assessment of childhood socioeconomic adversity took place. We therefore argue that earlier childhood depression may be on a causal pathway between exposure to early socioeconomic adversity and depression in young adulthood. It has been argued that adjusting for variables on a causal pathway will result in incorrect estimates of the total effect of the exposure on the outcome (Kumangar, 2012). In addition, a high degree of correlation between depressive symptoms at

10 years and 18 years may result in collinearity and over adjustment, whereby standard errors of estimates are very large and estimates of the effect of collinear variables are highly imprecise (Schisterman et al., 2009).

However, to reassure the Reviewer, we repeated our analyses adjusting the complete case model for the earliest measure of childhood depressive symptoms (SMFQ at 10 years) available in the ALSPAC. This extra adjustment did not change the conclusions of our previous analyses. There remained a strong evidence of an indirect pathway from early social adversity to diagnosed depression at 18 years via locus of control (β=0.099; 95% BC CI: 0.052 to 0.156, p<0.001). Given the argument outlined above and no changes to the conclusions of our original findings we did not amend the Manuscript to include the results of the additional adjustment.

Comment:

3. Third variables: as noted above, the authors focus on just one main link in the early-adversity-depression association, running via LoC. It seems quite plausible, however, that other factors will also be involved, and/or that the associations reported here might in practice reflect overlaps with other sources of influence. One that struck me - that would undoubtedly be associated with early adversity, and would likely be associated with both LoC and depression - is early cognitive skills, and particularly reading. ALSPAC undoubtedly includes measures of this kind, and it would I think be of value to assess their role in the current models - or again, at the least, to include them as potential confounders.

Response:

In line with recent methodological recommendations (Imai et al., 2010), our analyses adopted a statistical technique whereby assessment of mediation effects takes places within the context of possible mediator – outcome confounding (Muthén, 2011). Thus, we adjusted our analyses for variables that have been theoretically and empirically linked to socioeconomic adversity, LoC orientation and depression. Although socioeconomic adversity has been linked to more external LoC orientation, we are not aware of the longitudinal studies supporting the association between childhood cognitive skills (e.g., intellectual functioning, standardised math and reading scores, attention and memory) and LoC in adolescence. In addition, evidence with regard to the association between early cognitive skills and subsequent depression remains inconsistent and fraught with methodological issues

precluding from reliably inferring the direction of the effect (Castaneda et al., 2008). Although severe, persistent reading problems in childhood have been found to increase risk of depression in adolescence (Maughan et al., 2002), more longitudinal evidence is needed to support specific association between reading difficulties and psychopathology in community samples. Given this evidence, we concluded that there is not a sufficient theoretical and empirical basis to justify adjustment of our models for early childhood cognitive skills as possible confounders.

There is some longitudinal evidence to suggest that children with external LoC orientation have lower levels of school achievement (as measured by standardised math and reading scores) than children with internal orientation (Keith et al., 1986; von Stumm et al., 2009). Thus, it may be that childhood cognitive skills are on a causal pathway in the association between adolescent LoC and depression in young adulthood (i.e., explaining rather than confounding the association). This provides further support for our decision not to adjust the analyses for measures of childhood cognitive skills (please see our response to the Reviewer's previous comment).

Comment:

Presentational issues: in the Method section (p 8-9) the socio-economic adversity construct is described as derived from 14 binary indicators - though the account that follows only seems to mention six or so constructs. Figure 2 seems to refer to 16 indicators - and I assume from Figure 1 and Table 1 that the 14/16 constituent variables include repeated measures of a number of constructs. The account of the number of constituent indicators in the Method section needs to be clearer; I was also puzzled about the rationale for including eg 4 repeated indicators of home ownership but only two of low family income.

Response:

We thank the Reviewer for highlighting the inconsistencies in presenting information on early socioeconomic adversity. Figure 2 has now been amended to reflect 14 binary indicators (not 16) used to derive the latent construct of socioeconomic adversity. We have now clarified the Method section by stating that socioeconomic adversity indicators were assessed repeatedly from birth to 5 years. We also referred the readers to Figure 1 which clearly indicates the specific ages at which assessment of various indicators of socioeconomic adversity took place (please see *Method*, *Measures*, *Exposure: socioeconomic adversity*, p. 8). The Reviewer is right to assume that constituent variables are repeated measures of a

number of socioeconomic adversity constructs. The number of indicators at each time point vary due to data availability.

Reviewer #2: The manuscript entitled: 'Exposure to socioeconomic adversity in early life and risk of depression at 18 years: the mediating role of locus of control' (JAD-D-15-00248) reports findings from the large-scale study investigating the relationship between early socioeconomic adversity and depression in young adulthood. The study provides support for external locus of control mediating this relationship, so that socioeconomic adversity is associated with more external locus of control which in turn, is associated with higher risk for depression.

It is an interesting and very well-written manuscript, posing an important question that hasn't been thoroughly explained in the literature; this study could be of great interest to the JAD readers and wider research community. The authors took advantage of the longitudinal nature of the ALSPAC dataset and also provided a set of strong sensitivity analyses on missing data (which is essential in the light of a substantial sample drop in the current analyses).

However, I have a number of comments about the ms.

Comment:

Introduction

Introduction reads well, however a few clarifications may be needed. Specifically, a few additional examples of potential mechanisms linking socioeconomic adversity and depression could be helpful. This would further illustrate wider literature and a range of other potential mediators before focussing on locus of control.

Response:

In line with the Reviewer's suggestion, we have now provided additional examples of other potential mechanisms linking exposure to early childhood socioeconomic adversity to subsequent depression in adolescence and young adulthood (please see *Introduction*, p. 4, para. 2).

Comment:

Highlights provided are too repetitive and should focus on findings and their implications.

Response:

We have now focused our highlights on the main findings and their implications so that they are less repetitive (within the journal's word limit).

Comment:

Page 5 - explicit explanation of the direction of a social gradient in LoC is needed (i.e., low SES associated with more external LoC I presume?)

Response:

We have now explicitly stated the direction of the association between childhood social class and LoC (i.e., low childhood social class is associated with more external LoC; please see p. 5, para.3).

Comment:

Method/Results

Page 9 - a few examples of LoC questions could be helpful (at the authors' discretion)

Response:

The shortened version of the Nowicki-Strickland Internal-External scale (CNSIE; Nowicki & Strickland, 1973) is now included in the *Supplementary Material* submitted with the Manuscript.

Comment:

Page 15 - text referring to Table 4 (not 3)?

Response:

We thank the Reviewer for pointing out that text refers to Table 4 not Table 3. We have now amended accordingly.

Comment:

What is the rationale for socioeconomic adversity latent factor? Is it only for statistical reasons (i.e., normal distribution)? The idea of an underlying factor reflected in a range of observed socioeconomic indicators is not always welcome by research community.

Response:

We thank the Reviewer for this insightful comment. Indeed, combining different socioeconomic indicators into a composite measure has been previously criticised by the research community (Geyer et al. 2006). However, there was a good theoretical and statistical rationale to model indicators of socioeconomic disadvantage as a latent construct in our study. Theoretically, indicators used to derive a normally distributed latent factor of early adversity reflect aspects of the same underlying construct such as socioeconomic (e.g., maternal education, social class) and financial disadvantage (e.g., financial problems, material hardship, disposable income, and home ownership). Statistically, modelling early socioeconomic adversity as a normally distributed latent variable enabled us to capture persistent exposure across a range of indices of socioeconomic adversity from birth to 5 years, rather than estimating the effects of exposure to socioeconomic adversity at different time points. Other statistical advantage of latent variable approach includes better precision at modelling measurement error associated with assessment at multiple time points (Muthén, 2002).

Comment:

Discussion

Good discussion and coverage of potential neurobiological, psychological, and genetic mechanisms.

There's a substantial time gap between socioeconomic adversity and LoC - what could be the potential mechanism linking SES adversity and LoC? Such analyses may be beyond the scope of the manuscript but a brief discussion of more proximal factors that may explain the relationship would be useful. Knowing the dataset, there's potential for further serial mediation models which should be considered in the future.

Response:

The Reviewer is right to note that investigation of potential mechanisms that may explain the association between early socioeconomic adversity and LoC in adolescence is beyond the scope of this paper. However, we have now expanded the *Discussion* section to include a brief discussion of possible proximal mechanisms that could explain the aforementioned link (please see *Discussion*, *Alternative mechanisms*, p.18, para. 2). We have also suggested that additional studies which test complex mediational models are warranted to provide further

insights into multiple pathways between socioeconomic adversity, LoC and depression. This opens up avenues for future research including that with the ALSPAC data.

Comment:

Finally, the manuscript should be proofread for typos and appropriate referencing style.

Response:

The manuscript has been fully proofread for typos and referencing style in accordance with the journal's requirements.

Comment:

Tables/Figures

Figure 2 - I would recommend graphically differentiating significant and non-significant pathways. The label should state whether these are standardized estimates.

Response:

As recommended by the Reviewer, Figure 2 now graphically differentiates significant and non-significant pathways. We have also clearly indicated that path coefficients on the edges are unstandardised regression coefficients (due to the well-defined metrics of our exposure and outcome variables; Preacher & Hayes, 2008).

References

Carton, J. S., Nowicki S., 1994. Antecedents of individual differences in locus of control of reinforcement. Genet. Soc. Gen. Psych. 3, 23-58.

Castaneda, A. E., Tuulio-Henriksson, A., Marttunen, M., Suvisaari, J., Lönnqvist, J. 2008. A review on cognitive impairments in depressive and anxiety disorders with a focus on young adults. J. Affect. Disorders 106, 1-27.

Geyer, S., Hemstrom, O., Peter, R., Vagero, D. 2006. Education, income, and occupational class cannot be used interchangeably in social epidemiology. Empirical evidence against a common practice. J. Epidemiol. Commun. H. 9, 804-810.

Imai, K., Keele, L., Yamamoto, T., 2010. Identification, inference and sensitivity analysis for causal mediation effects. Stat. Sci. 25, 51-71.

Keith, T. Z., Pottebaum, S. M., & Eberhart, S. 1986. Effects of self-concept and locus of control on academic achievement: A large-sample path analysis. J. Psychoeduc. Assess. 4, 61-72.

Kliewer, W., Sandler, I. S., 1992. Locus of control and self-esteem as moderators of stressor-symptom relations in children and adolescents. J. Abnorm. Child Psych. 20, 394-413.

Kulas, H., 1996. Locus of control in adolescence: a longitudinal study. Adolescence 31, 721-729.

Kamangar, F. 2012. Confounding variables in epidemiologic studies: basics and beyond. Arch Iran Med, 158, 508-516.

Maughan, B., Rowe, R., Loeber, R., Stouthamer-Loeber, M. 2003. Reading problems and depressed mood. J. Abnorm. Child Psych. 31, 219-229.

Muthén, B. O. 2002. Beyond SEM: General latent variable modeling. Behaviormetrika 29, 81-117.

Preacher, K. J., Hayes, A. F. 2008. Asymptotic and resampling strategies for assessing and comparing indirect effects in multiple mediator models. Behav. Res. Methods 40, 879-891.

Rothman, K. J., Greenland, S., Lash. T. L. 2008. Modern epidemiology. Lippincott Williams & Wilkins: Philadelphia, PA.

Schisterman, E. F., Cole, S. R., Platt, R. W. 2009. Overadjustment bias and unnecessary adjustment in epidemiologic studies. Epidemiology 20, 488-495.

Skinner, E. A., Connell, J. P., 1986. Control understanding: suggestions for a developmental framework, in: Baltes, M. M., Baltes, P. B. (Eds)., The psychology of aging and control. Erlbaum: Hillsdale, NJ.

von Stumm, S., Gale, C. R., Batty, G. D., & Deary, I. J. 2009. Childhood intelligence, locus of control and behaviour disturbance as determinants of intergenerational social mobility: British Cohort Study 1970. Intelligence 37, 329-340.

*Manuscript
Click here to view linked References

Exposure to socioeconomic adversity in early life and risk of depression at 18 years: the mediating role of locus of control

Iryna Culpin PhD [a], Lexine Stapinski PhD [a,b], Ömür Budanur Miles PhD [c], Ricardo Araya PhD MRCPsych [d], Carol Joinson PhD [a]

[a] School of Social and Community Medicine, University of Bristol, Bristol, UK

[b] Centre of Research Excellence in Mental Health and Substance Use, University of New South Wales, Sydney, Australia.

[c] Child and Adolescent Mental Health Service, St David's Hospital Cardiff, Cwm Taf Health Board, Cardiff, Wales, UK

[d] London School of Hygiene & Tropical Medicine, Faculty of Epidemiology and Public Health, London, UK.

Word count: 4,935

Corresponding author: Iryna Culpin, School of Social and Community Medicine, University of Bristol, Barley House, Oakfield Grove, Clifton, BS82BN.

Email: Iryna.Culpin@bristol.ac.uk; Phone: (0117) 331 0162; Fax: (0117) 954 4026

ABSTRACT

Background: Previous studies have linked exposure to early socioeconomic adversity to depression, but the mechanisms of this association are not well understood. Locus of control (LoC), an individual's control-related beliefs, has been implicated as a possible mechanism, however, longitudinal evidence to support this is lacking.

Methods: The study sample comprised 8,803 participants from a UK cohort, the Avon Longitudinal Study of Parents and Children (ALSPAC). Indicators of early socioeconomic adversity were collected from the antenatal period to 5 years and modelled as a latent factor. Depression was assessed using the Clinical Interview Schedule-Revised (CIS-R) at 18 years. LoC was assessed with the Nowicki-Strickland Internal-External (CNSIE) scale at 16 years.

Results: Using structural equation modelling, we found that 34% of the total estimated association between early socioeconomic adversity and depression at 18 years was explained by external LoC at 16 years. There was weak evidence of a direct pathway from early socioeconomic adversity to depression after accounting for the indirect effect via external locus of control. Socioeconomic adversity was associated with more external LoC, which, in turn, was associated with depression.

Limitations: Attrition may have led to an underestimation of the direct and indirect effect sizes in the complete case analysis.

Conclusions: Results suggest that external LoC in adolescence is one of the factors mediating the link between early adversity and depression at 18 years. Cognitive interventions that seek to modify maladaptive control beliefs in adolescence may be effective in reducing risk of depression following early life adversity.

Keywords: Avon Longitudinal Study of Parents and Children (ALSPAC); early childhood; socioeconomic adversity; depression; locus of control.

1. Introduction

An increasing body of research supports the association between early socioeconomic adversity and risk for depression in adolescence and young adulthood (Chapman et al., 2004; Kessler et al., 2010; Patten et al., 2014). In particular, socioeconomic disadvantage, poverty, inadequate housing, and residential instability during early childhood have been linked to later depression (Gilman et al., 2003; Gilman et al., 2002). However, little is known about the psychological mechanisms underlying this association (Grant, 2006). Increased knowledge of factors explaining the link between aspects of early socioeconomic adversity and increased risk of depression could provide insights into potentially modifiable targets for intervention.

Depression is a complex disorder and a number of risk factors and causal mechanisms (e.g., psychosocial, neurocognitive, gene-environment interplay) are likely to be involved (Maughan et al., 2013). Early socioeconomic adversity could exert a direct effect on depression via biological systems, such as the hypothalamic pituitary axis (HPA), and these effects could be independent of exposure to adulthood adversity (Stansfeld et al., 2011). Alternatively, early socioeconomic disadvantage may set off a chain of proximal psychosocial events and individual characteristics that increase the risk for depression, such as adverse family processes (e.g., negative parenting; Conger et al., 2002), environmental stressors (e.g., inadequate schooling; Barrera et al., 2002), and maladaptive coping styles and cognitive attributions (Paschall & Hubbard, 1998). Specifically, exposure to early socioeconomic adversity may lead to a pattern of psychological vulnerability characterised by maladaptive perceptions of the self and life events that increase risk for depression (Hammen, 2005). One aspect of psychological vulnerability that could be influential in the link between childhood adversity and depression is adolescents' internal *versus* external control-related beliefs also known as locus of control (LoC; Rotter, 1996; Strickland, 1989).

It has been argued that an individual's beliefs related to their perceived sense of control over their environment relate to their psychological well-being and mental health outcomes (Chorpita & Barlow, 1998; Shapiro et al., 1993). Individuals are thought to differ in the extent to which they perceive themselves as being able to control life events through their efforts and actions (internal LoC), or that life events are controlled by external forces such as luck, chance and powerful others (external LoC; Rotter, 1966). Although antecedents of LoC in adolescence are not fully understood, it has been suggested that early experiences of adverse and uncontrollable events, including persistent exposure to socioeconomic disadvantage, may foster external LoC orientation characterised by diminished sense of perceived control over one's life and environment (Bryant & Trockel, 1976; Chorpita, 2001; Gilman et al., 2003). Children and adolescents who develop external LoC and experience uncertainty about the extent of control they have over life events have also been hypothesised to be at increased risk of developing depression (Chorpita, 2001; Ostander & Herman, 2006).

Socioeconomic differences in the sense of personal control have been examined in early cross-sectional studies (Lachman & Weaver, 1998), indicating that those in more disadvantaged groups (characterised by lower income and less education) have lower sense of control and are more likely to believe in the role of external forces and powerful others (Bosma et al., 1999; Lachman & Weaver, 1998). However, longitudinal evidence linking early socioeconomic adversity and adolescent LoC orientation is lacking. Similarly, numerous cross-sectional but few longitudinal studies have examined the link between LoC orientation and depression. Consistently, an external LoC has been found to be associated with depression in childhood (Cole et al., 2001), adolescence (Donnelly, 1999; Muris et al., 2004) and adulthood (Benassi et al., 1988). However, prospective longitudinal studies examining the association between external LoC and depression are limited (Harrow et al., 2009; Frenkel et al., 1995) and further investigations are warranted.

Studies that examine LoC as a possible pathway in the early adversity – depression association are also scarce and not without limitations. The majority of studies are cross-sectional and rely on retrospective assessment of childhood adversity and LoC, thus precluding inferences about the temporal relationship between experiences of adversity, LoC orientation and depression (Deardoff et al., 2003; Kim et al., 1997; Sandler et al., 2000). Furthermore, these studies focus on examining the possible mediating role of LoC in specific samples of children such as those from divorced, bereaved or severely disadvantaged families (Deardoff et al., 2003; Haine et al., 2003; Kim et al., 1997; Sandler et al., 2000). Other limitations include overreliance on measures based on a single reporter (e.g., adolescent self-reports; Kim et al., 1997), composite measures of stress (e.g., total number of various negative life events; Kim et al., 1997), and lack of diagnostic measures of depression (Deardoff et al., 2003). Thus, there is need for prospective studies to examine possible mediating role of LoC in the association between exposure to various aspects of early socioeconomic adversity and depression in young adulthood.

Family adverse experiences are multifaceted and dynamic. Thus, it is important to control for possible confounders whilst examining the pathways between early socioeconomic adversity, LoC and depression in young adulthood. For instance, indices of socioeconomic disadvantage, such as poverty, often co-occur with parental depression and negative parental cognitions (Edwards et al., 2003; Dong et al., 2004), and these events are associated with both development of external LoC orientation and depression in young adulthood. Epidemiological evidence has long established a strong link between socioeconomic disadvantage in childhood and an increased risk of a psychiatric disorder, including depression (Solantaus et al., 2004). Paternal depression, like maternal depression, may compromise parenting behaviours and have an adverse impact on the way parents interact with their children (Lyons-Ruth et al., 2002; Paulson et al., 2006) who are more

likely to develop external LoC. For instance, aspects of parental cognition, especially maternal warmth and acceptance, have been linked to internal LoC orientation in children and are considered to be antecedents of LoC (Carton & Nowicki; 1996; Muris & Meesters, 2004).

The current study, using data from the Avon Longitudinal Study of Parents and Children (ALSPAC), examines whether LoC mediates the association between early socioeconomic adversity and later depression. It has been previously demonstrated in this cohort that aspects of childhood adversity such as victimisation (e.g., bullying) and harsh parenting are associated with more external LoC orientation (Fisher et al., 2013), and other studies have also reported an association between exposure to socioeconomic adversity and an increased risk of depression (Joinson *et al., in press*). We hypothesised that exposure to socioeconomic adversity from birth to 5 years will be associated with more external LoC orientation at 16 years and that this would constitute an indirect pathway between early adversity and increased risk of depression at 18 years. We used structural equation modelling (SEM) to test the hypothesised model using a latent factor to encapsulate exposure to early socioeconomic adversity during the first 5 years of life, and by adjusting the model for a range of child and parental confounders.

2. Method

2.1. Participants

The sample comprised participants from the Avon Longitudinal Study of Parents and Children (ALSPAC), an ongoing UK population-based study. The study website contains details of all data that is available through a searchable data dictionary (http://www.bris.ac.uk/alspac/researchers/data-access/data-dictionary). Ethical approval for the study was obtained from the ALSPAC Ethics and Law Committee and the Local Research Ethics Committees. We restricted our sample to participants recruited during Phase

I in order to include covariate information collected during early infancy (this data is not available for Phase II participants). During Phase I enrolment, 14,541 pregnant mothers residing in the former Avon Health Authority in the south-west of England with expected dates of delivery between 1 April 1991 and 31 December 1992 were recruited to the study. These pregnancies resulted in 14,062 live births, of which 13,617 singletons were alive at 1 year of age. For further details on the cohort profile, representativeness and phases of recruitment see (Boyd et al., 2013).

2.2. Measures

2.2.1. Exposure: socioeconomic adversity

We used 14 binary indicators derived from questionnaires administered to mothers in the antenatal period and during the first 5 years of the study child's life to derive a normally distributed latent factor of socioeconomic adversity (Figure 1). The variables assessed in the antenatal period were: maternal educational attainment classified as none/minimal (mothers with the lowest level of qualifications generally obtained at age 16 years, vocational qualifications, or none) versus higher-level qualifications (mothers with ordinary-level qualifications generally obtained at age 16 years/advanced-level qualifications generally obtained at age 18 years/university degree); social class assessed on the basis of the lower of the mother's or partner's occupational social class using the 1991 British Office of Population and Census Statistics classification and dichotomised into social class I-IV (professional, managerial, or skilled professionals) and V-VI (partly skilled or unskilled occupations); and financial problems (occurrence of major financial problems versus none). The following socioeconomic adversity indicators were assessed repeatedly from birth to 5 years (Figure 1): financial problems (yes/no); home ownership defined as living in owner-occupier or privately rented accommodation versus subsidised housing; material hardship derived using a cut-off of ≥ 5 corresponding to material hardship scores in the top 20% of the

sample; and low family disposable income derived from a continuous weekly income measure and dichotomised to comprise those who were in the lowest income quartile *versus* the rest of the sample.

2.2.2. Outcome: depression

Depression was assessed using the Clinical Interview Schedule – Revised (CIS-R; Lewis, 1994) at a research clinic attended at mean age 17.8 years (hereafter referred to as 18 years). Participants completed a self-administered computerised version of the CIS-R, which measures current symptoms across multiple domains. Computer algorithms were used to identify psychiatric disorders according to DSM-IV and ICD-10 diagnostic criteria (Lewis, 1994). The CIS-R is designed for, and has been widely used with community samples in the UK and elsewhere (e.g., Clark et al., 2007; Jenkins et al., 1997). Good agreement has been demonstrated between administration by a clinically trained interviewer, lay interviewer and self-administration using the computerised version (Lewis, 1994). Based on this interview we derived a binary variable to indicated presence *versus* absence of a depressive disorder.

2.2.3. Mediator: locus of control

Adolescents completed a 12-item shortened version of the Nowicki-Strickland Internal-External scale (CNSIE; Nowicki 1976; Nowicki & Duke, 1974; Nowicki & Strickland, 1973) (see *Supplementary Material*) as part of face-to-face clinic assessment at age 16 (median age at completion=16.7; inter-quartile range=16.6-16.10). A person with a higher ‘internal’ score on this measure is considered to perceive that the outcome of events is under their own control, whilst a person with a higher ‘external’ score on this measure is considered to perceive that the outcome of events is controlled by outside circumstances. A total score was derived by summing scores for all the items, with higher scores indicating a more external LoC. The questionnaire has been shown to have good construct validity and test-retest reliability in children from ages 9 through 18 years (Nowicki, 1976; Nowicki &

Duke, 1974; Nowicki & Strickland, 1973) and has been used extensively in previous research (Nowicki & Duke, 2013). For participants missing responses to one or two locus of control items (e.g., less than 20% of the total scale items), unanswered questions were replaced with the mean of the participants' own responses to the rest of the scale items.

2.2.4. Confounding variables

Child's gender, maternal and paternal depressive symptoms and maternal cognitive style were included as potential confounders as they have been previously shown to be associated with exposure to childhood adversity, locus of control and depression (Edwards et al., 2003; Klein et al., 2005; Paulson et al., 2006; Solantaus et al., 2004). Maternal and paternal self-reported depression was assessed using the Edinburgh Postnatal Depression Scale (EPDS; Cox et al., 1987) administered when the study child was 8 months. Maternal cognitive style was assessed using a 6-item scale (Evans et al., 2005) derived from a broader measure of intrapersonal sensitivity (Boyce & Parker, 1989) and administered at 18 weeks gestation. The six items comprising the scale map onto negative cognitions outlined in Beck's cognitive theory of depression (e.g., 'I always expect criticism'; see Evans et al., 2005). The scores from the six items were summed up to derive a total negative cognitive style score (range = 0-18), with higher scores reflecting more negative cognitions.

2.3. Statistical analyses

Descriptive statistics were obtained using STATA 12.0. Primary analyses were conducted using Mplus software version 7.11 (Muthén & Muthén, 2012) using the WLSMV estimator. Prior to examining the hypothesised mediation pathway, we first tested a measurement model incorporating the outcome (binary variable indicating diagnosis of depression), exposure (latent factor of early socioeconomic adversity), hypothesised mediator (continuous locus of control score) and all potential confounders (child's sex, maternal cognitive style, maternal and paternal depression). The measurement model is illustrated in

Figure 1. Early socioeconomic adversity was estimated as a latent variable comprising the 14 binary indicators of socioeconomic disadvantage described above. Residual variances of the repeated early adversity indicators were allowed to co-vary to accommodate common method variance at each assessment. The exposure variable and potential confounders were also free to co-vary. Acceptability of the model fit was evaluated using standard goodness of fit indices. The chi-square test of exact fit is stringent and sensitive to sample size with simulations showing the test will routinely reject good models when sample size is large (e.g., n > 200; Brown, 2006; Schumacker & Lomax, 2010), thus we considered several relative fit indices. A root mean square error of approximation (RMSEA) value less than 0.06, Tucker-Lewis index (TLI) and comparative fit index (CFI) values greater than 0.95 are considered indicative of good fit to the data (Hu & Bentler, 1999).

Once the measurement model had been confirmed, we tested a structural model to estimate the direct and indirect pathways of interest. As techniques to assess mediation progress, methodologists have emphasised the importance of considering the potential impact of mediator-outcome confounders within mediation models (e.g., Imai et al., 2010). Thus, we adopted the mediation approach recommended by Muthén (2011), which allows for the assessment of mediation effects within the context of potential mediator-outcome confounders. Using the “Model Constraint” command, new parameters and standard errors representing causally-defined direct and indirect effects (Robins & Greenland, 1992; Valeri & VanderWeele, 2013) were calculated from model estimated parameters. For a detailed description and Mplus input syntax see (Muthén, 2011). First we estimated an unadjusted mediation model that included only the exposure (early socioeconomic adversity), mediator (locus of control at 16 years) and outcome (diagnosed depression at 18 years). Next we estimated the model adjusted for the child’s sex. The final model was adjusted for the child’s sex and potential maternal and paternal confounders. We calculated bootstrapped standard

errors and confidence intervals from 1,000 bootstrap samples (MacKinnon et al., 2004) to account for non-normality associated with a binary outcome.

2.4. Missing data

Complete-case analyses can be biased if data are not missing completely at random. In order to examine the impact of response attrition on our conclusions, we examined characteristics of the complete-case sample compared with the rest of the ALSPAC cohort. We used STATA 12.0 to impute 50 datasets, each entailing 20 cycles of regression switching, using multiple imputation by chained equations (Royston, 2009). This is a recommended procedure for missing data (Sterne et al., 2009) which assumes data are missing at random (MAR) conditional on the variables in the imputation model. Our imputation model included a number of auxiliary socio-demographic and mental health variables predictive of incomplete variables and/or missingness, including locus of control score at 8 years and depressive symptoms scores from ages 10 through 19 years. In order to ensure plausibility of the MAR assumption, cases were included in the imputation sample only if data were available for depressive symptoms and each socioeconomic adversity indicator on at least one measurement occasion. Therefore we imputed data for a sample of 6,851 participants. Predictive mean matching was employed for non-normal variables (White et al., 2011). The imputed data was imported into Mplus and mediation analyses were repeated over the 50 imputed datasets combining the estimates according to Rubin's rules (Royston et al., 2009).

3. Results

3.1. Descriptive characteristics

The starting sample was 8,803 participants for whom data were available on each socioeconomic adversity indicator at one or more time points. Of these, 3,528 participants (40.1%) completed the CIS-R diagnostic interview at 18 years, and 4,074 participants

(46.3%) completed the locus of control measure at 16 years. Complete data for the exposure (socioeconomic adversity), outcome (diagnosed depression) and mediator (locus of control) were available for 2,663 participants. For 1,892 of these participants, complete data were also available for all potential confounders. Given the considerable response attrition, sensitivity analyses on multiply imputed data examined the impact of attrition on our conclusions. Descriptive characteristics for the complete case sample compared to partial responders is provided in Table 1. Participants with complete data came from more socially advantaged families with fewer depressive symptoms.

3.2. Measurement model

The measurement model incorporating early adversity, locus of control and potential confounders is shown in Figure 1. Fit statistics indicated that the measurement model fit the data well (RMSEA=0.04, 95% CI: 0.03 to 0.04; TFI=0.97; CFI=0.97). This supported the adequacy of the model for subsequent tests of structural paths and mediation.

3.3. Association between early socioeconomic adversity, locus of control and depression

Prior to examining the hypothesised mediation pathway, we examined the univariable associations between socioeconomic adversity, LoC and depression. There was evidence that greater early socioeconomic adversity was associated with an increased risk of depression at 18 years (β=0.191; 95% BC CI: 0.055 to 0.340, p=0.007). There was also evidence that more external locus of control at 16 years was associated with increased risk of diagnosed depression at 18 years (β=0.105; 95% BC CI: 0.069 to 0.136, p<0.001). In order to assist interpretation of the size of these probit estimates, Table 2 shows the predicted probability of depression diagnosis at different levels (± 1 and 2 standard deviations) of early socioeconomic adversity and locus of control. Experiences of socioeconomic adversity were associated with LoC orientation (β=0.752; 95% BC CI: 0.583 to 0.973, p<0.001).

3.4. Mediation Model

A series of models were estimated to assess the hypothesised mediation pathway. Table 3 shows parameter estimates, bootstrapped standard errors and bias-corrected (BC) confidence intervals for the unadjusted and adjusted models. Within the unadjusted model, there was strong evidence of an indirect pathway from early social adversity to diagnosed depression at 18 years via locus of control (β=0.123; 95% BC CI: 0.073 to 0.185, p<0.001). There was weak evidence of a direct pathway from early social adversity to diagnosed depression once the indirect effect via locus of control was accounted for (β=0.216; 95% BC CI: -0.008 to 0.484, p=0.088).

Adjustment for child's sex (adjusted model 1) and maternal and paternal characteristics (adjusted model 2) made little difference to the parameter estimates. Within the fully adjusted model, there remained strong evidence of an indirect path from early social adversity through locus of control to diagnosed depression at 18 years (β=0.128; 95% BC CI: 0.073 to 0.195, p<0.001). This indirect path via locus of control accounted for 34% of the total estimated association between early socioeconomic adversity and diagnosed depression. Path estimates for the fully adjusted mediation model are illustrated in Figure 2. There was strong evidence that child's sex was also associated with diagnosed depression (β=0.815; 95% BC CI: 0.516 to 1.156, p<0.001), as well as locus of control at 16 years (β=0.410; 95% BC CI: 0.255 to 0.549, p<0.001). The direction of these associations indicates that females were more likely to be diagnosed with depression, and report more external locus of control. There was also some evidence to suggest that offspring of fathers with higher depression reported more external locus of control at 16 years (β=0.033; 95% BC CI: 0.004 to 0.061, p=0.021).

3.5. Missing data: sensitivity analyses

In order to examine the impact of response attrition on our findings, mediation analyses were repeated using 50 imputed datasets for a sample of 6,851 participants. Results

from these analyses are presented in Table 4. The resulting fraction of missing information (FMI) estimates (Schafer, 1997) indicated that 50 imputed datasets were sufficient. The results from analyses with imputed data supported our findings: the direct and indirect effect estimates were in the same direction and led to the same overarching conclusions. However, the sizes of the observed direct and indirect effects were greater in the imputed data. Although it is not possible to entirely account for the impact of response attrition, the pattern of missing data and analyses suggest that attrition lead to an underestimation of the direct and indirect effects size in the complete case analysis. This was most apparent for the remaining direct effect from early socioeconomic adversity to diagnosed depression. Within the fully adjusted analysis using imputed data, the indirect path from early socioeconomic adversity to diagnosed depression through locus of control was estimated as β=0.192 (p < 0.001), while the remaining direct pathway from early socioeconomic adversity to depression was estimated as β= 0.522 (p=0.002). Based on analyses using imputed data, we would estimate a slightly lower proportion (27%) of the total association between early socioeconomic adversity and depression is accounted for by the indirect path through locus of control.

4. Discussion

4.1. Main findings

We examined whether exposure to early socioeconomic adversity is associated with LoC in adolescence and a diagnosis of depression at 18 years. We further investigated whether LoC mediates the association between socioeconomic adversity and depression in young adulthood. We found evidence that exposure to early socioeconomic adversity is associated with more external LoC orientation at 16 years, which, in turn, is associated with depression at 18 years. This finding is consistent with previous research linking external LoC

to depression (Harrow et al., 2009; Twenge et al., 2004) and highlights important contributions of perceived sense of control in development of depression. Approximately 34% (27% in analyses with imputed data) of the total estimated association between socioeconomic adversity and diagnosed depression was accounted for by the indirect path through external LoC in the model adjusted for child's gender and various parental characteristics. This finding is consistent with studies supporting the mediating role of LoC in the association between exposure to early adversity and depression in young adulthood (Deardorff et al., 2003; Hunter et al., 2010; Kliewer & Sandler, 1992). Although attenuated, there was evidence of a direct pathway from early socioeconomic adversity to depression once the indirect effect via locus of control was accounted for, suggesting an independent effect of early adversity on development of depression.

4.2. Strength and limitations

The current study has several strengths, including a longitudinal design, a large community-based sample, a measure of clinical diagnosis of depression as an outcome, and adjustment for a range of confounders. To our knowledge, no previous prospective longitudinal study have examined LoC orientation as a mechanism of the association between early socioeconomic adversity and depression in young adulthood. Modelling early socioeconomic adversity as a latent variable enabled us to capture exposure to various indices of socioeconomic adversity from birth to 5 years. A limitation of the study relates to sample attrition, which is strongly associated with socioeconomic disadvantage in the ALSPAC and this has important implications for the internal validity of the study. In particular, participants from lower socioeconomic background and those with mental health problems were underrepresented in our sample. However, the attrition rates in this cohort are similar to those observed in other large-scale longitudinal studies (Callaway et al., 2007). The pattern of

missing data and results of the sensitivity analyses suggest that attrition lead to an underestimation of the direct and indirect effect sizes in the complete case analysis. Repeating the analyses with the imputed sample adjusted for the bias introduced by missing data and improved efficiency compared to complete case analysis (Klebanoff & Cole, 2008; Spratt et al., 2010).

4.3. Alternative mechanisms

A proportion of the association between early socioeconomic adversity and depression was not explained by external LoC orientation. This finding is in line with previous studies examining LoC as a pathway between childhood adversity and mental health problems (Fisher et al., 2013). This could indicate direct traumatic effect of exposure to childhood adversity on subsequent development of depression in young adulthood via biological systems such as the hypothalamic pituitary axis (HPA; Penza et al., 2003). Indeed, accumulating evidence suggest that childhood adversity is associated with HPA dysregulation and heightened stress reactivity in adolescents and adults (Heim et al., 2000; McLaughlin et al., 2009), which, in turn, may lead to maladaptive emotional and social functioning. Similarly, there is evidence to suggest that individuals with external LoC tend to display heightened neuroendocrine and autonomic stress responsiveness (Declerck et al., 2006; Steptoe & Willemsen, 2004), whereas individuals with internal LoC show lower cortisol responses to stress (Pruessner et al., 1997). Although there is no longitudinal research to support this assumption, it could be that dysregulation of HPA axis is a common neurobiological mechanism linking early life adversity, development of maladaptive control-related beliefs and depression.

The unexplained proportion of the association between socioeconomic adversity and depression via external LoC could also be due to other mediating factors or residual

confounding not accounted for in the present analyses. It has been suggested that the link between early life adversity and negative mental health outcomes in adulthood, including depression, could be explained by low self-esteem, interpersonal difficulties, and maladaptive coping strategies (Whiffen & MacIntosh, 2005). In addition, experiences of poverty and material hardship in childhood often co-occur with emotional and physical neglect, abuse and victimisation, which are, in turn, strong predictors of adolescent and adulthood depression (Turner et al., 2006). Our assessment of early life adversity did not include questions on more severe forms such as abuse and other trauma, thus, possible mediating effect of these factors could not be examined in this study. In addition, individual sense of control is believed to be a complex, multi-dimensional construct better conceptualised as a combination of LoC, self-efficacy, learned helplessness, and an individual's desire of control (Shapiro et al., 1993). Examination of this multidimensional concept of control beliefs was beyond the scope of our study.

Mechanisms that could explain the link between experiences of early socioeconomic adversity and external LoC also warrant further examination. It has been suggested that early experiences of poverty may foster external LoC orientation in children through exposure to parental depression and negative parenting (Chorpita, 2001; Gilman et al., 2003). Indeed, there is some longitudinal evidence to support a link between negative parenting practices and external LoC orientation (Muris et al., 2004), whilst parental warmth has been linked to a more internal LoC (Carton & Nowicki, 1994). Additional studies which test complex mediational models are warranted to provide further insights into multiple pathways between early socioeconomic adversity, LoC and depression.

Although the study controlled for a range of prospectively measured parental and child characteristics, we did not examine possible genetic confounders that may explain

observed associations. Genetic influences explain approximately 40% to 50% of the variance in depression (Levinson, 2006), however, there is little evidence on the heritability of LoC. The few studies that examined genetic influences on individual differences in LoC estimate these to be between 10% and 55% (Johansson et al., 2001). It is, therefore, possible that the association between external LoC and depression may be partly explained by common genes that contribute to both. Results from future genetically informative designs may provide further insights towards understanding of the mechanisms underlying this association.

4.4. Other associations of interest

Other associations of interest emerged in the context of the present study. Consistent with numerous epidemiological research, girls in our sample were more likely to meet criteria for a depression diagnosis (Parker & Brotchie, 2010) and reported more external LoC (Feingold, 1994) than boys. Gender differences in depression are well-documented with differences in cognitive functioning and more frequent exposure to adverse experiences in childhood often cited as contributing factors (Piccinelli & Wilkinson, 2000). There is also some longitudinal evidence to suggest that girls move toward more external LoC disposition during middle adolescence, whilst boys become more internal (Kulas, 1996; Ross & Mirowsky, 2002). However, these findings are inconsistent and require further longitudinal research for adequate replication. Interestingly, the findings of this study suggest more external LoC orientation in offspring of depressed fathers. Research on parental depression supports the importance of studying fathers in relation to child outcomes (Ramchandani et al., 2009). Paternal depression is associated with more parent–child conflict (Kane & Garber, 2004) and harsh disciplining (Schacht et al., 2009), which, in turn, has been linked to more external LoC in children and adolescents (Lynch et al., 2002). Although mother–child conflict may be more frequent than father–child conflict, it has been suggested that the latter

may be more harmful to children's behavioural and emotional development (Forehand et al., 1987). Thus, the strong influence of paternal depression and associated coercive parenting could explain this finding.

4.5. Clinical implications

The findings of the present study have important implications for depression prevention programs. Although LoC is thought to be a relatively enduring individual characteristic (Kulas, 1996), it has been suggested that it is amenable to psychological interventions, particularly in childhood and adolescence (deCharms, 1976; Trice, 1990). Evidence indicates that programs focusing on restructuring cognitive coping strategies and control-related beliefs result in shifts in LoC from less external to more internal orientation (Figurelli & Hartman, 1994). Internal LoC, in turn, is associated with better adherence to treatment (Steel et al., 2000) and favourable therapy outcomes (Delsignore & Schnyder, 2007; Weisz, 1986). Our findings suggest that depression prevention programs should include a component that addresses cognitive beliefs about control because shifting external LoC orientation to internal could help to reduce the risk of developing depression.

Role of funding source

The UK Medical Research Council, the Wellcome Trust (Grant ref.: 092731) and the University of Bristol provide core support for ALSPAC.

Conflict of interest

No conflict declared.

Acknowledgement

We thank all of the families who took part in this study, the midwives for their help in recruiting the participants, and the whole ALSPAC team, which includes interviewers, computer and laboratory technicians, clerical workers, research scientists, volunteers, managers, receptionists and nurses. This publication is the work of the authors and they will serve as guarantors for the content of this paper.

References

Barrera, M., Prelow, H. M., Dumka, L. E., Gonzales, N. A., Knight, G. P., Michaels, M. L., Roosa, M. W., Tein, J., 2002. Pathways from family economic conditions to adolescents' distress: Supportive parenting, stressors outside the family, and deviant peers. J. of Community Psychol. 30, 135-152.

Benassi, V. A, Dufour, C. L, Sweeney, P. D., 1988. Is there a relationship between locus of control orientation and depression? J. Abnorm. Psychol. 3, 357-367.

Bosma, H., van de Mheen, H. D., Mackenbach, J. P., 1999. Social class in childhood and general health in adulthood: questionnaire study of contribution of psychological attributes. Brit. Med. J. 318, 18-22.

Boyce, P., Parker, G., 1989. Development of a scale to measure interpersonal sensitivity. Aust. NZ J. Psychiat. 23, 341-351.

Boyd, A., Golding, J., Macleod, J., Lawlor, D. A., Fraser, A., Henderson, J., Molloy, L., Ness, A., Ring, S., Smith, G. D., 2013. Cohort Profile: The ‘Children of the 90s’—the index offspring of the Avon Longitudinal Study of Parents and Children. Int. J. Epidemiol. 42, 111-127.

Brown, T. A., 2006. Confirmatory factor analysis for applied research. Guilford Press, New York.

Bryant, B. K., Trockel, J. F., 1976. Personal history for psychological stress related to locus of control orientation among college women. J. Consult. Clin. Psych. 44, 266.

Callaway, L. K., McIntyre, H. D., O'Callaghan, M., Williams, G. M., Najman, J. M., Lawlor, D. A., 2007. The association of hypertensive disorders of pregnancy with weight gain

over the subsequent 21 years: findings from a prospective cohort study. A. J. Epidemiol. 166, 421-428.

Carton, J. S., Nowicki Jr, S., 1996. Origins of generalized control expectancies: Reported child stress and observed maternal control and warmth. J. Soc. Psychol. 136, 753-760.

Carton, J. S., Nowicki S., 1994. Antecedents of individual differences in locus of control of reinforcement. Genet. Soc. Gen. Psych. 3, 23-58.

Chapman, D.P., Whitfield, C.L., Felitti, V.J., Dube, S.R., Edwards, V.J., Anda, R.F., 2004. Adverse childhood experiences and the risk of depressive disorders in adulthood. J. Affect. Disorders 82, 217-225.

Chorpita, B. F., 2001. Control and development of negative emotion, in: Vasey, M. W., Dadds, M. R. (Eds.), The developmental psychopathology of anxiety. Oxford University press, New York.

Chorpita, B. F., Barlow, D. H., 1998. The development of anxiety: the role of control in the early environment. Psychol. Bull. 124, 3-21.

Clark, C., Rodgers, B., Caldwell, T., Power, C., Stansfeld, S., 2007. Childhood and adulthood psychological ill health as predictors of midlife affective and anxiety disorders: the 1958 British Birth Cohort. Arch. Gen. Psychiat. 64, 668-678.

Cole, D. A., Jacquez, F. M., Maschman, T. L., 2001. Social origins of depressive cognitions: A longitudinal study of self-perceived competence in children. Cognitive Ther. Res. 25, 377-395.

Conger, R. D., Wallace, L. E., Sun, Y., Simons, R. L., McLoyd, V. C., Brody, G. H., 2002. Economic pressures in African American families: a replication and extension of the family stress model. Dev. Psychol. 38, 179-193.

Cox, J., Holden, J., Sagovsky, R., 1987. Detection of postnatal depression. Development of the 10-item Edinburgh Postnatal Depression Scale. Brit. J. Psychiat. 150, 782-786.

Deardorff, J., Gonzales, N. A., Sandler, I. N., 2003. Control beliefs as a mediator of the relation between stress and depressive symptoms among inner-city adolescents. J. Abnorm. Child Psychol. 31, 205-217.

deCharms, R., 1976. Enhancing motivation. Irvington: New York.

Declerck, C. H., Boone, C., De Brabander, B., 2006. On feeling in control: a biological theory for individual differences in control perception. Brain and Cognition 62, 143-176.

Delsignore, A., Schnyder, U., 2007. Control expectancies as predictors of psychotherapy outcome: A systematic review. Brit. J. Clin. Psychol. 46, 467-483.

Dong, M., Anda, R. F., Felitti, V. J., Dube, S. R., Williamson, D. F., Thompson, T. J., Loo, C. M., Giles, W. H., 2004. The interrelatedness of multiple forms of childhood abuse, neglect, and household dysfunction. Child Abuse Neglect 28, 771-784.

Donnelly, M., 1999. Factors associated with depressed mood among adolescents in Northern Ireland. J. Community Appl. Soc. 9, 47-59.

Edwards, V. J., Holden, G. W., Felitti, V. J., Anda, R. F., 2003. Relationship between multiple forms of childhood maltreatment and adult mental health in community

respondents: results from the adverse childhood experiences study. Am. J. Psychiat. 160, 1453-1460.

Evans, J., Heron, J., Lewis, G., Araya, R., Wolke, D., 2005. Negative self-schemas and the onset of depression in women: longitudinal study. Brit. J. Psychiat. 186, 302-307.

Feingold, A., 1994. Gender differences in personality: a meta-analysis. Psychol. Bull. 116, 429-456.

Figurelli, G. A., & Hartman, B. W., 1994. Assessment of change in scores on personal control orientation and use of drugs and alcohol of adolescents who participate in a cognitively oriented pretreatment intervention. Psychol. Rep. 75, 939-944.

Fisher, H. L., Schreier, A., Zammit, S., Maughan, B., Munafò, M. R., Lewis, G., Wolke, D., 2013. Pathways between childhood victimization and psychosis-like symptoms in the ALSPAC birth cohort. Schizophrenia Bull. 39, 1045-1055.

Forehand, R., McCombs, A., Brody, G. H., 1987. The relationship between parental depressive mood states and child functioning. Adv. Behav. Res. Ther. 9, 1-20.

Frenkel, E., Kugelmass, S., Nathan, M., & Ingraham, L. J., 1995. Locus of control and mental health in adolescence and adulthood. Schizophrenia Bull. 21, 219-226.

Gilman, S. E., Kawachi, I., Fitzmaurice, G. M., Buka, S. L., 2003. Socio-economic status, family disruption and residential stability in childhood: relation to onset, recurrence and remission of major depression. Psychol. Med. 33, 1341-1355.

Gilman, S. E., Kawachi, I., Fitzmaurice, G. M., Buka, S. L., 2002. Socioeconomic status in childhood and the lifetime risk of major depression. Int. J. Epidemiol. 31, 359-367.

Grant, K. E., Compas, B. E., Thurm, A. E., McMahon, S. D., Gipson, P. Y., Campbell, A. J., Krochock, K., Westerholm, R. I. 2006. Stressors and child and adolescent psychopathology: Evidence of moderating and mediating effects. Clin. Psychol. Rev. 26, 257-283.

Grant, K. E., Compas, B. E., Stuhlmacher, A. F., Thurm, A. E., McMahon, S. D., Halpert, J. A., 2003. Stressors and child and adolescent psychopathology: moving from markers to mechanisms of risk. Schizophrenia Bull. 129, 447-466.

Haine, R. A., Ayers, T. S., Sandler, I. N., Wolchik, S. A., & Weyer, J. L., 2003. Locus of control and self-esteem as stress-moderators or stress-mediators in parentally bereaved children. Death Stud. 27, 619-640.

Hammen, C., 2005. Stress and depression. Annu. Rev. Clin. Psych. 1, 293-319.

Harrow, M., Hansford, B. G., Astrachan-Fletcher, E. B., 2009. Locus of control: relation to schizophrenia, to recovery, and to depression and psychosis: a 15-year longitudinal study. Psychiat. Res. 168, 186-192.

Heim, C., Newport, D. J., Heit, S., Graham, Y. P., Wilcox, M., Bonsall, R., Miller, A. H., Nemeroff, C. B., 2000. Pituitary-adrenal and autonomic responses to stress in women after sexual and physical abuse in childhood. J. Am. Med. Assn. 284, 592-597.

Hu, L. T., Bentler, P. M., 1999. Cutoff criteria for fit indexes in covariance structure analysis: Conventional criteria versus new alternatives. Structural Equation Modeling 6, 1-55.

Hunter, S. C., Durkin, K., Heim, D., Howe, C., Bergin, D., 2010. Psychosocial mediators and moderators of the effect of peer-victimization upon depressive symptomatology. J. Child Psychol. Psyc. 51, 1141-1149.

Imai, K., Keele, L., Yamamoto, T., 2010. Identification, inference and sensitivity analysis for causal mediation effects. Stat. Sci. 25, 51-71.

Jenkins, R., Lewis, G., Bebbington, P., Brugha, T., Farrell, M., Gill, B., Meltzer, H., 1997. The National Psychiatric Morbidity Surveys of Great Britain: initial findings from the Household Survey. Psychol. Med. 27, 775-789.

Johansson, B., Grant, J. D., Plomin, R., Pedersen, N. L., Ahern, F., Berg, S., McClearn, G. E., 2001. Health locus of control in late life: A study of genetic and environmental influences in twins aged 80 years and older. Health Psychol. 20, 33-40.

Joinson, C., Kounali, D., Lewis, G. A prospective cohort study of family socioeconomic position at birth and onset of depressive symptoms and depression. *In press.*

Kane, P., Garber, J., 2004. The relations among depression in fathers, children's psychopathology, and father–child conflict: A meta-analysis. Clin. Psychol. Rev. 24, 339-360.

Kessler, R. C., McLaughlin, K. A., Green, J. G., Gruber, M. J., Sampson, N. A., Zaslavsky, A. M. et al., 2010. Childhood adversities and adult psychopathology in the WHO World Mental Health Surveys. Brit. J. Psychiat. 197, 378-385.

Kim, L. S., Sandler, I. N., Tein, J. Y., 1997. Locus of control as a stress moderator and mediator in children of divorce. J. Abnorm. Child Psych. 25, 145-155.

Klebanoff, M. A., Cole, S. R., 2008. Use of multiple imputation in the epidemiologic literature. Am. J. Epidemiol. 168, 355-357.

Klein, D. N., Lewinsohn, P. M., Rohde, P., Seeley, J. R., Olino, T. M., 2005. Psychopathology in the adolescent and young adult offspring of a community sample of mothers and fathers with major depression. Psychol. Med. 35, 353-365.

Kliewer, W., Sandler, I. N., 1992. Locus of control and self-esteem as moderators of stressor-symptom relations in children and adolescents. J. Abnorm. Child Psych. 20, 393-413.

Kulas, H., 1996. Locus of control in adolescence: a longitudinal study. Adolescence 31, 721-729.

Lachman, M. E., Weaver, S. L., 1998. The sense of control as a moderator of social class differences in health and well-being. J. Pers. Soc. Psychol. 74, 763-773.

Levinson, D. F., 2006. The genetics of depression: a review. Biol. Psychiat. 60, 84-92.

Lewis, G., 1994. Assessing psychiatric disorder with a human interviewer or a computer. *Journal of Epidemiology and Community Health, 48*, 207-210.

Lynch, S., Hurford, D. P., Cole, A., 2002. Parental enabling attitudes and locus of control of at-risk and honors students. Adolescence 37, 527-549.

Lyons-Ruth, K., Wolfe, R., Lyubchik, A., Steingard, R., 2002. Depressive symptoms in parents of children under age 3: Sociodemographic predictors, current correlates, and associated parenting behaviors, in: Halfon, N., McLearn, K. T. (Eds.), Child rearing in America: Challenges facing parents with young children. Cambridge: New York.

MacKinnon, D. P., Lockwood, C. M., Williams, J., 2004. Confidence Limits for the Indirect Effect: Distribution of the Product and Resampling Methods. Multivar. Behav. Res. 39, 99-128.

Maughan, B., Collishaw, S., Argyris, A., 2013. Depression in childhood and adolescence. J. Can. Academy Child Adolescent Psychiat. 21, 35-40.

McLaughlin, K. A., Hatzenbuehler, M. L., Hilt, L. M., 2009. Emotion dysregulation as a mechanism linking peer victimization to internalizing symptoms in adolescents. J. Consult. Clin. Psych. 77, 894.

Muris, P., Meesters, C., Schouten, E., Hoge, E., 2004. Effects of perceived control on the relationship between perceived parental rearing behaviors and symptoms of anxiety and depression in nonclinical preadolescents. J. Youth Adolescence 33, 51-58.

Muthén, B., 2011. Applications of causally defined direct and indirect effects in mediation analysis using SEM in Mplus. *Manuscript submitted for publication*.

Muthén, L. K., Muthén, B. O., 2012. Mplus User's Guide: Seventh Edition. Muthén & Muthén: Los Angeles, CA.

Nowicki, S., 1976. Factor structure of locus of control in children. J. Genet. Psychol. 129, 13-17.

Nowicki, S., Duke, M. P., 2013. The Nowicki-Strickland life-span locus of control scales: Construct validation, in: Lefcourt, H. M. (Eds.), Research with the locus of control construct, Volume 2: Developments and Social Problems. Academic Press, Inc: New York.

Nowicki, S., Duke, M. P., 1974. A preschool and primary internal-external control scale. Dev. Psychol. 10, 874-881.

Nowicki, S., Strickland, B., 1973. A locus of control scale for children. *Journal of Consulting & Clinical Psychology, 40*, 148-154.

Ostrander, R., Herman, K. C., 2006. Potential cognitive, parenting, and developmental mediators of the relationship between ADHD and depression. J. Consult. Clin. Psych. 74, 89-98.

Parker, G., Brotchie, H., 2010. Gender differences in depression. Int. Rev. Psychiat. 22, 429-436.

Paschall, M. J., Hubbard, M. L., 1998. Effects of neighbourhood and family stressors on African American male adolescents' self-worth and propensity for violent behavior. J. Consult. Clin. Psych. 66, 825-831.

Patten, S. B., Wilkes, T. C. R., Williams, J. V. A., Lavorato, D. H., El-Guebaly, N., Schopflocher, D. et al., 2014. Retrospective and prospectively assessed childhood adversity in association with major depression, alcohol consumption and painful conditions. Epidemiol. Psychiat. Sciences 1-8.

Paulson, J. F., Dauber, S., Leiferman, J. A., 2006. Individual and combined effects of postpartum depression in mothers and fathers on parenting behavior. Pediatrics 118, 659-668.

Penza, K. M., Heim, C., Nemeroff, C. B., 2003. Neurobiological effects of childhood abuse: implications for the pathophysiology of depression and anxiety. Arch. Women's Mental Health 6, 15-22.

Piccinelli, M., Wilkinson, G., 2000. Gender differences in depression: critical review. Brit. J. Psychiat. 177, 486-492.

Pruessner, J. C., Gaab, J., Hellhammer, D. H., Lintz, D., Schommer, N., Kirschbaum, C., 1997. Increasing correlations between personality traits and cortisol stress responses obtained by data aggregation. Psychoneuroendocrino. 22, 615-625.

Ramchandani, P., Psychogiou, L., 2009. Paternal psychiatric disorders and children's psychosocial development. Lancet, 374, 646-653.

Robins, J. M., Greenland, S., 1992. Identifiability and exchangeability for direct and indirect effects. Epidemiology 3, 143-155.

Ross, C. E., Mirowsky, J., 2002. Age and the gender gap in the sense of personal control. Soc. Psychol. Quart. 125-145.

Rotter, J.B., 1966. Generalised expectancies for internal versus external control of reinforcement. Psychol. Monographs 8, 37.

Royston, P., 2009. Multiple imputation of missing values: Further update of ice, with an emphasis on categorical variables. Stata J. 9, 466-477.

Royston, P., Carlin, J. B., White, I. R., 2009. Multiple imputation of missing values: new features for mim. Stata J. 9, 252-264.

Sandler, I. N., Kim-Bae, L. S., MacKinnon, D., 2000. Coping and negative appraisal as mediators between control beliefs and psychological symptoms in children of divorce. J. Clin. Child Psychol. 29, 336-347.

Schacht, P. M., Cummings, E. M., Davies, P. T., 2009. Fathering in family context and child adjustment: a longitudinal analysis. J. Fam. Psychol. 23, 790-797.

Schafer, J. L., 1997. Analysis of Incomplete Multivariate Data. Chapman & Hall: New York.

Shapiro JR, D. H., Blinder, B. J., Hagman, J., Pituck, S., 1993. A psychological "sense-of-control" profile of patients with anorexia nervosa and bulimia nervosa. Psychol. Rep. 73, 531-541.

Schumacker, R. E., Lomax, R. G., 2010. A beginner's guide to structural equation modeling, third ed. Routledge/Taylor & Francis Group, US: New York.

Solantaus, T., Leinonen, J., Punamäki, R. L., 2004. Children's mental health in times of economic recession: replication and extension of the family economic stress model in Finland. Dev. Psychol. 40, 412-429.

Spratt, M., Carpenter, J., Sterne, J. A., Carlin, J. B., Heron, J., Henderson, J., & Tilling, K., 2010. Strategies for multiple imputation in longitudinal studies. Am. J. Epidemiol. 172, 478-487.

Stansfeld SA, Clark C, Rodgers B., Caldwell T., Power C., 2011. Repeated exposure to socioeconomic disadvantage and health selection as life course pathways to mid-life depressive and anxiety disorders. Soc. Psych. Psych. Epid. 46, 549-558.

Steel, Z., Jones, J., Adcock, S., Clancy, R., Bridgford-West, L., Austin, J., 2000. Why the high rate of dropout from individualized cognitive-behavior therapy for bulimia nervosa? Int. J. Eat. Disorder. 28, 209-214.

Steptoe, A., Willemsen, G., 2004. The influence of low job control on ambulatory blood pressure and perceived stress over the working day in men and women from the Whitehall II cohort. J. Hypertens. 22, 915-920.

Sterne, J. A. C., White, I. R., Carlin, J. B., Spratt, M., Royston, P., Kenward, M. G., Wood, A. M., Carpenter, J. R., 2009. Multiple imputation for missing data in epidemiological and clinical research: potential and pitfalls. Brit. Med. J. 338.

Stricland, B.R., 1989. Internal-external control expectancies: from contingency to creativity. Am. Psychol. 44, 1-12.

Trice, A. D., 1990. Adolescents' locus of control and compliance with contingency contracting and counselling interventions. Psychol. Rep. 67, 233-234.

Twenge, J. M., Zhang, L., Im, C., 2004. It's beyond my control: a cross-temporal meta-analysis of increasing externality in locus of control, 1960-2002. Pers. Soc. Psychol. Rev. 8, 308-319.

Turner, H. A., Finkelhor, D., Ormrod, R., 2006. The effect of lifetime victimization on the mental health of children and adolescents. Soc. Sci. Med. 62, 13-27.

Valeri, L., VanderWeele, T. J., 2013. Mediation analysis allowing for exposure–mediator interactions and causal interpretation: Theoretical assumptions and implementation with SAS and SPSS macros. Psychol. Methods 18, 137.

Weisz, J. R., 1986. Contingency and control beliefs as predictors of psychotherapy outcomes among children and adolescents. J. Consult. Clin. Psych. 54, 789.

Whiffen, V. E., MacIntosh, H. B., 2005. Mediators of the link between childhood sexual abuse and emotional distress a critical review. Trauma, Violence, & Abuse 6, 24-39.

White, I. R., Royston, P., Wood, A. M., 2011. Multiple imputation using chained equations: Issues and guidance for practice. Stat. Med. 30, 377-399.

Table(s)

Table 1

Individual and family characteristics for the complete sample and partial responders

	Initial ALSPAC Sample (n=13,617)		
	Complete case (n=2,663)	Partial Data (n=10,954)	Statistical Test[a]
Categorical measures	(%)	(%)	*Chi*^{2}
Female	58.2	46.0	$\chi^2(1) = 128.0^{**}$
Maternal Education			
Degree	22.9	10.0	$\chi^2(3) = 610.4^{**}$
Advanced High School	29.3	20.6	
Standard High School	33.2	35.0	
No High School	14.7	34.4	
Parental Social Class			
I Professional	20.8	11.0	$\chi^2(4) = 322.7^{**}$
II Managerial/Technical	46.6	40.2	
III Skilled Non-Manual	21.9	26.7	
IV Skilled Manual	7.6	15.2	
IV & V: Partly or Unskilled	3.0	6.8	
Low Family Income			
Child Age 3 years	11.2	22.5	$\chi^2(1) = 144.4^{**}$
Child Age 4 years	14.8	28.9	$\chi^2(1) = 186.6^{**}$
Does Not Own Home			
Child Age 8 months	10.3	26.9	$\chi^2(1) = 307.5^{**}$
Child Age 2 years	10.7	25.4	$\chi^2(1) = 243.6^{**}$
Child Age 3 years	10.6	24.1	$\chi^2(1) = 207.6^{**}$
Child Age 5 years	9.8	22.7	$\chi^2(1) = 193.9^{**}$
Major Financial Problems			
Child Age 8 months	11.9	15.7	$\chi^2(1) = 22.2^{**}$
Child Age 2 years	12.2	15.8	$\chi^2(1) = 20.2^{**}$
Child Age 3 years	12.9	16.5	$\chi^2(1) = 18.2^{**}$
Child Age 4 years	10.3	13.4	$\chi^2(1) = 16.3^{**}$
Material Hardship			

Child Age 8 months	22.6		31.2		$\chi^2(1) = 71.5^{**}$	
Child Age 2 years	20.2		29.7		$\chi^2(1) = 86.8^{**}$	
Child Age 3 years	19.7		27.4		$\chi^2(1) = 59.9^{**}$	
Child Age 4 years	15.0		20.9		$\chi^2(1) = 40.9^{**}$	
Offspring Depression Diagnosis 18 years	7.1		9.1[1]		$\chi^2(1) = 5.5^{**}$	
Continuous Measures	Mean	*SD*	Mean	*SD*	Mean diff	95% CI
Offspring Locus of control 16 years	3.0	(2.0)	3.4	(2.2)	0.42	0.30 -0.54
Maternal Cognitive Style	4.9	(3.4)	5.0	(3.7)	0.13	-0.3-0.28
Maternal Depression	4.9	(4.4)	5.6	(4.8)	0.67	0.46-0.87
Paternal Depression	3.2	(3.5)	3.4	(3.8)	0.22	0.02-0.41

[a] Differences in sample characteristics according to response attritions were tested using chi-square tests for categorical variables and *t* tests for continuous variables. $^{**}p < 0.001$.

Note. ALSPAC Avon Longitudinal Study of Parents and Children.

Table 2

Estimated prevalence of CIS-R depression diagnosis at varying levels of early social adversity and locus of control.

	Estimated prevalence (%) of depression				
Predictor variable	-2 *SDs*	-1 *SDs*	Mean	+1 *SDs*	+2 *SDs*
Socioeconomic Adversity (Latent Factor)	4.1	5.3	6.9	8.9	11.2
Locus of Control (Total Score)	1.3	2.1	3.5	5.4	8.1

Note. This table shows estimated prevalence of diagnosed depression at 1 and 2 standard deviations above and below the mean for each predictor variable. Estimates are derived from univariable model with no other confounders included.

CIS-R Clinical Interview Schedule – Revised.

Table 3

Estimates Using Complete Case Data (N = 2,663)[a] of the Direct and Indirect Effects of Early Socioeconomic Adversity on Depression Diagnosis at 18 Years Mediated Through Locus of Control at 16 Years.

	Model Estimates			
	β	*SE*	*p*	BC 95% CI[b]
Unadjusted Model				
1. Total effect<br>Early adversity on depression diagnosis at 18	0.339	0.124	0.006	0.098-0.598
2. Indirect effect<br>Early adversity on depression diagnosis at 18, through locus of control at 16	0.123	0.027	<0.001	0.073-0.185
3. Remaining direct effect<br>Early adversity on depression diagnosis at 18, adjusted for locus of control	0.216	0.127	0.088	-0.008-0.484
Adjusted 1 (Gender)				
1. Total effect<br>Early adversity on depression diagnosis at 18	0.386	0.152	0.011	0.096-0.692
2. Indirect effect<br>Early adversity on depression diagnosis at 18, through locus of control at 16	0.135	0.032	<0.001	0.074-0.204
3. Remaining direct effect<br>Early adversity on depression diagnosis at 18, adjusted for locus of control	0.251	0.155	0.104	-0.023-0.577
Adjusted 2 (Gender, Maternal & Paternal Factors)				
1. Total effect<br>Early adversity on depression diagnosis at 18	0.377	0.156	0.016	0.074-0.675
2. Indirect effect<br>Early adversity on depression diagnosis at 18, through locus of control at 16	0.128	0.030	<0.001	0.073-0.195
3. Remaining direct effect<br>Early adversity on depression diagnosis at 18, adjusted for locus of control	0.249	0.158	0.114	-0.032-0.574

[a] Analyses restricted to participants with complete mediator (locus of control) and outcome (depression diagnosis) data.

[b] BC 95% CI: bias corrected (1,000 bootstrap samples)

Table 4

Estimates Using Imputed Data (N = 6,851) of the Direct and Indirect Effects of Early Socioeconomic Adversity on Depression Diagnosis at 18 Years Mediated Through Locus of Control at 16 Years.

	Model Estimates			
	β	*SE*	*p*	FMI[a]
Unadjusted Model				
1. Total effect<br>Early adversity on depression diagnosis at 18	0.584	0.120	<0.001	0.330
2. Indirect effect<br>Early adversity on depression diagnosis at 18, through locus of control at 16	0.152	0.032	<0.001	0.389
3. Remaining direct effect<br>Early adversity on depression diagnosis at 18, adjusted for locus of control	0.433	0.124	<0.001	0.337
Adjusted 1 (Gender)				
1. Total effect<br>Early adversity on depression diagnosis at 18	0.581	0.121	<0.001	0.328
2. Indirect effect<br>Early adversity on depression diagnosis at 18, through locus of control at 16	0.149	0.032	<0.001	0.390
3. Remaining direct effect<br>Early adversity on depression diagnosis at 18, adjusted for locus of control	0.432	0.125	0.001	0.336
Adjusted 2 (Gender, Maternal & Paternal Factors)				
1. Total effect<br>Early adversity on depression diagnosis at 18	0.714	0.166	<0.001	0.429
2. Indirect effect<br>Early adversity on depression diagnosis at 18, through locus of control at 16	0.192	0.042	<0.001	0.389
3. Remaining direct effect<br>Early adversity on depression diagnosis at 18, adjusted for locus of control	0.522	0.172	0.002	0.342

[a] FMI: Fraction of Missing Information

Figure(s)

Fig. 1. Measurement model of the hypothesised associations between socioeconomic adversity in early life, locus of control and depression diagnosis at 18 years, adjusted for potential confounders.

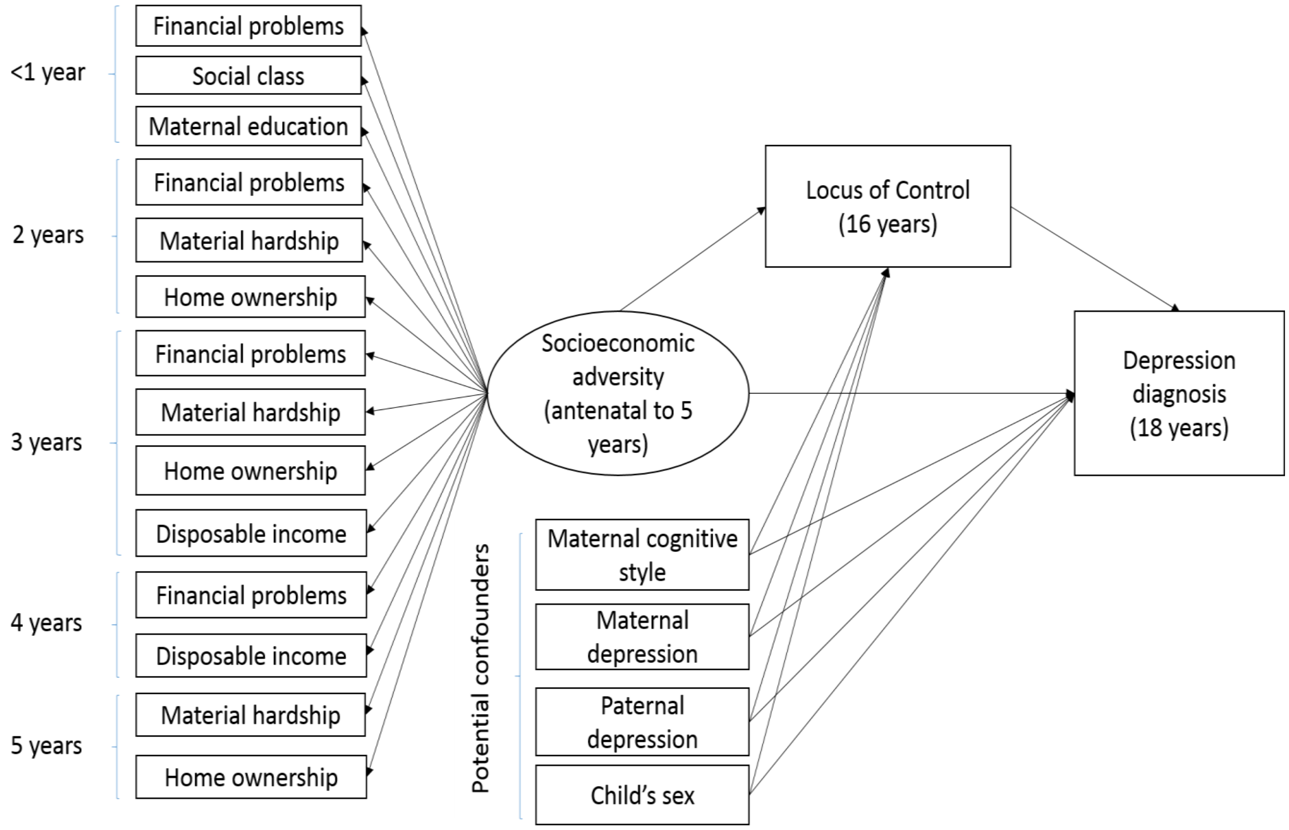

Note. Observed variables are represented by squares, whilst the latent variable is represented by circle. Covariances are not shown to reduce figure complexity.

Fig. 2. Structural mediation model estimating the direct and indirect pathways from early social adversity to diagnosed depression at 18 years, adjusted for potential confounders (n=2,663).

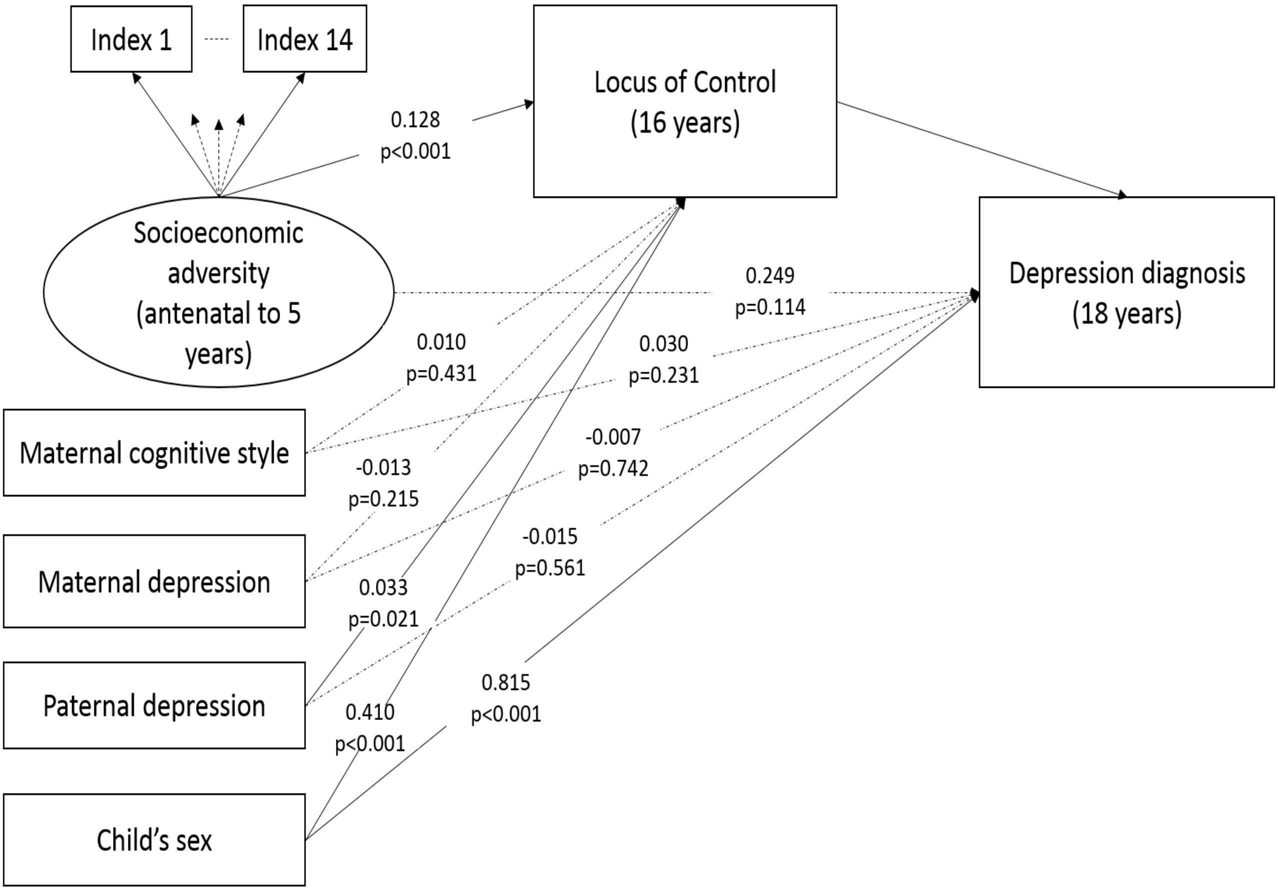

Note. Path coefficients on the edges are unstandardised regression estimates. Pathways delineated as dash lines are statistically non-significant (p>0.05).

Acknowledgement

We thank all of the families who took part in this study, the midwives for their help in recruiting the participants, and the whole ALSPAC team, which includes interviewers, computer and laboratory technicians, clerical workers, research scientists, volunteers, managers, receptionists and nurses. This publication is the work of the authors and they will serve as guarantors for the content of this paper.

Conflict of interest

All authors declare that they have no conflicts of interest.

*Contributors
Click here to download Contributors: Contributors.doc

Contributors

I. Culpin wrote the manuscript under the supervision of C. Joinson. L. Stapinski analysed the data. I. Culpin and L. Stapinski interpreted the findings. R. Araya and Ö. Miles commented on drafts of the manuscript and revised it critically for intellectual content. All authors were involved in editing the manuscript and have approved the final manuscript prior to submission.

Role of the funding source

The UK Medical Research Council, the Wellcome Trust (Grant ref.: 092731) and the University of Bristol provide core support for ALSPAC.

Highlights

- We examine locus of control as a pathway between social adversity and depression.
- External locus of control mediates the link between social adversity and depression.
- Social adversity is linked to more external locus of control and depression.
- More external locus of control is associated with higher risk of depression.
- Depression prevention programs should address cognitive beliefs about control.

Supplementary Material

Shortened version of the Nowicki-Strickland Internal-External scale (CNSIE; Nowicki & Strickland, 1973)

Question		
1.	Do you feel that wishing can make good things happen?	Yes/No
2.	Are people nice to you *no matter what you do*?	Yes/No
3.	Do you *usually* do badly in your school work even when you try hard?	Yes/No
4.	When a friend is angry with you is it hard to make that friend like you again?	Yes/No
5.	Are you surprised when your teacher praises you for your work?	Yes/No
6.	When bad things happen to you is it *usually* someone else's fault?	Yes/No
7.	Is doing well in your class-work just a matter of 'luck' for you?	Yes/No
8.	Are you *often* blamed for things that just aren't your fault?	Yes/No
9.	When you get into an argument or fight is it *usually* the other person's fault?	Yes/No
10.	Do you think that preparing for tests is a waste of time?	Yes/No
11.	When nice things happen to you is it usually because of 'luck'?	Yes/No
12.	Does planning ahead make good things happen?	Yes/No
